# Characterization of two polyvalent phages infecting *Enterobacteriaceae*

**DOI:** 10.1038/srep40349

**Published:** 2017-01-16

**Authors:** Sana Hamdi, Geneviève M. Rousseau, Simon J. Labrie, Denise M. Tremblay, Rim Saïed Kourda, Karim Ben Slama, Sylvain Moineau

**Affiliations:** 1 Laboratoire des Microorganismes et Biomolécules Actives, Faculté des Sciences de Tunis, Université de Tunis El Manar, 2092 Tunis, Tunisie; 2 Institut Supérieur des Sciences Biologiques Appliquées de Tunis, Université de Tunis El Manar, 2092 Tunis, Tunisie; 3 Département de Biochimie, de Microbiologie, et de Bio-informatique and PROTEO, Faculté des Sciences et de Génie, Félix d’Hérelle Reference Center for Bacterial Viruses, and GREB, Faculté de Médecine Dentaire, Université Laval, Québec City, Québec, Canada

## Abstract

Bacteriophages display remarkable genetic diversity and host specificity. In this study, we explore phages infecting bacterial strains of the *Enterobacteriaceae* family because of their ability to infect related but distinct hosts. We isolated and characterized two novel virulent phages, SH6 and SH7, using a strain of *Shigella flexneri* as host bacterium. Morphological and genomic analyses revealed that phage SH6 belongs to the *T1virus* genus of the *Siphoviridae* family. Conversely, phage SH7 was classified in the *T4virus* genus of the *Myoviridae* family. Phage SH6 had a short latent period of 16 min and a burst size of 103 ± 16 PFU/infected cell while the phage SH7 latent period was 23 min with a much lower burst size of 26 ± 5 PFU/infected cell. Moreover, phage SH6 was sensitive to acidic conditions (pH < 5) while phage SH7 was stable from pH 3 to 11 for 1 hour. Of the 35 bacterial strains tested, SH6 infected its *S. flexneri* host strain and 8 strains of *E. coli*. Phage SH7 lysed additionally strains of *E. coli* O157:H7, *Salmonella* Paratyphi, and *Shigella dysenteriae.* The broader host ranges of these two phages as well as their microbiological properties suggest that they may be useful for controlling bacterial populations.

Bacteriophages, the viruses of bacteria, are characterized by their nanometer size as well as their obligatory parasitism. They are present in the same ecological niche as their hosts and contribute to bacterial ecology and evolution^1^,^2^. The vast majority of phages also possess a proteinaceous tail (*Caudovirales* order) that enables the specific recognition and subsequent adsorption to a receptor at the surface of the host bacterium. The *Caudovirales* are further divided into three families: *Myoviridae* (long contractile tail), *Siphoviridae* (long noncontractile tail) and *Podoviridae* (short tail). In the past decade, comparative genomic and proteomic analyses have been essential in revealing the diversity and evolutionary relationships between phages as well as the levels of interaction with their bacterial hosts. A better understanding of these bacterial viruses has led to several prospective biotechnological applications. For example, virulent phages have potential for preventing or treating bacterial disease^3^,^4^, detecting bacteria^5^, and decontaminating surfaces^6^,^7^.

Phages are usually species-specific and even strain-specific^8^, however, some polyvalence is observed, predominantly among phages of Enterobacteria^9^,^10^ and staphylococci^11^. These polyvalent phages are able to infect strains from either different genera or species^12^.

Members of the *Enterobacteriaceae* family are also closely related and are sometimes taxonomically difficult to distinguish^13^,^14^. The ability of these polyvalent lytic phages to infect various host species makes host range an imperfect indicator of phage relatedness. Therefore, phages of the same morphological family are grouped in genera/species based on similarities in genome sequences^15^.

Phages with broad host ranges are most often isolated from natural microbial communities that promote genetic exchanges^16^,^17^. Under selective pressure, tail fiber genes appear to evolve faster than other phage genes, presumably because it seems advantageous to gain new specificities to infect different hosts as well as to enter other ecological niches^1^,^18^. The *T4virus* genus is one of the best-characterized phage groups for which tail fibers have been extensively studied and shown relative rapid adaptation. They have extended host ranges, mostly due to their unique ability to recognize various host receptors (outer membrane protein or lipopolysaccharide (LPS)) using the same tail fiber protein (gp37^T4^)^19^. Members of this genus have high genetic identity and the modest differences observed are related to their adaptation to novel host constraints^20^,^21^. For example, coliphages T4 and AR1 share a highly conserved core genome but have distinct host ranges^22^. Phage T4 infects *E. coli* B and K-12 but not *E. coli* O157:H7, while AR1 is very specific to strains of *E. coli* O157:H7^23^. Another example was observed in the *T1virus* genus of the *Siphoviridae* family. Coliphage Rtp is closely related to coliphage T1 but acquired a unique tail tip that allows it to recognize a different host receptor. Phage T1 uses the FhuA outer membrane protein as a receptor and requires TonB for infection while phage Rtp uses LPS^24^.

Because of the renewed interest in lytic phages as biocontrol agents, new phages are sought after as they offer the possibility of increasing bacterial strain coverage in the design and development of phage cocktails^11^,^25^. In an effort to isolate new phages effective against several strains, we investigated phages infecting the *Enterobacteriaceae* family. From sewage samples in Tunisia, we isolated two virulent phages that belong to the *Myoviridae* and *Siphoviridae* families. Their genomic, proteomic and microbiological characteristics provided an overview of their evolutionary relationships with other previously characterized phages and suggest that they may have potential to control bacterial populations.

## Results

### Isolation of bacteria and phages

A Gram-negative bacterial isolate, SF1, was recovered from a wastewater sample in Tunisia. The 16S rRNA sequencing and MLST analysis revealed that this strain belongs to the *Shigella flexneri* species with the sequence type ST216. This strain was used as the host organism to search for virulent phages. Phages were isolated from water samples of the wadi of Chotrana in Tunisia. Two plaques with distinct morphology were picked at random and single-plaque purified at least three times. These phage isolates were named SH6 and SH7. Phage SH6 formed clear plaques of 2 mm in diameter after only 3 hours of incubation at 37 °C. Phage SH7 formed very small plaques of around 0.2 mm in diameter after overnight incubation at 37 °C.

### Host range and microbiological properties

The host range of phages SH6 and SH7 was then tested on 35 bacterial strains by spot test of diluted phage lysate (Table 1). Among the 35 strains tested, phage SH7 was able to infect 27 bacterial strains while phage SH6 infected 9 strains. Both isolated phages infected their host strain *S. flexneri*, the host strain of the T-odd and T-even coliphages, *E. coli* B11303, and 7 other *E. coli* K-12 derived strains. Phage SH7 also infected 16 strains of the Shiga toxin-producing *E. coli* O157:H7, one strain of *Salmonella* Paratyphi, and one strain of *Shigella dysenteriae*. For phage SH6, a lysis zone was observed at 10^0^ and 10^−1^ with the 16 strains of *E. coli* O157:H7 but they were likely the result of lysis from without (at high multiplicity of infection) as no clear plaques could be observed or propagated.

A one-step growth curve experiment using their host strain *S. flexneri* SF1 revealed that phage SH6 has a short latent period of only 16 min and a burst size of 103 ± 16 PFU/infected cell. The latent period of phage SH7 was 23 min with a burst size of 26 ± 5 PFU/infected cell. The pH sensitivity of the two phages was determined by exposing them for one hour at 37 °C to pH ranging from 2 to 11. Phage SH6 was sensitive to acidic conditions as it was inactivated when exposed to pH 2 and pH 3. It also had a 2-Log reduction at pH 4 but was stable from pH 5 to pH 11. Phage SH7 was stable from pH 3 to 11 and inactivated only at pH 2.

### Morphological analysis

**Figure 1:**
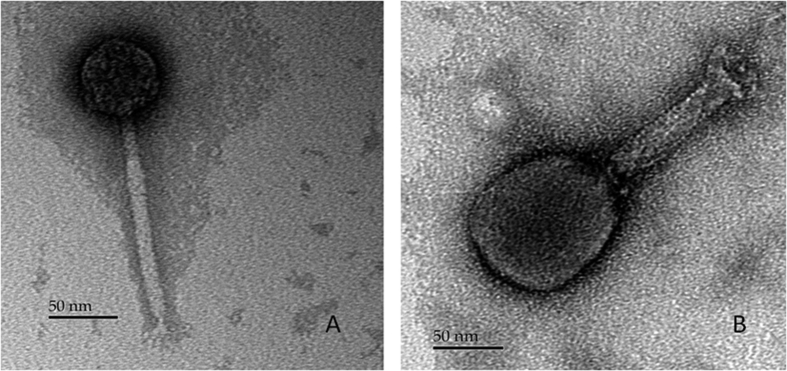
Electron micrographs of phage SH6 (**A**) and phage SH7 (**B**).

Electron microscopy observations of purified phage SH6 revealed an icosahedral capsid of 62 ± 2 nm in diameter with a long noncontractile flexible tail of 161 ± 2 nm in length by 13 ± 1 nm in width, morphology indicative of the *Siphoviridae* family (Fig. 1A). Phage SH7 had an elongated capsid of 112 ± 5 nm in length by 89 ± 3 nm in width and a long contractile tail of 116 ± 4 nm in length by 22 ± 2 nm in width. SH7 belongs to the *Myoviridae* family (Fig. 1B).

### Proteomic analysis

The purified phages SH6 and SH7 were analyzed by liquid chromatography/tandem mass spectrometry (LC-MS/MS). The LC-MS/MS analysis detected 11 structural proteins in phage SH6 with a sequence coverage ranging from 14 to 55% (Table 2). These proteins included the portal protein, capsid protein, tail proteins, tail tape measure protein, tail fiber proteins and four other proteins of unknown function. Analysis of phage SH7 by LC-MS/MS detected 13 structural proteins with a sequence coverage ranging from 4 to 31% including proteins of the capsid, tail, tail fibers, neck and procapsid core. Other than the phage SH7 structural proteins, we also identified one ADP-ribosylase protein with the lowest coverage (3%, 76 kDa), which corresponds to an enzyme that is usually not present in the phage structure. The target of the phage ADP-ribosylase is the host RNA polymerase. The ADP-ribosylation of RNA polymerase is involved in increasing gene expression after infection but it is not essential for phage development^26^,^27^. While it is unclear if this is a contaminant of the phage purification process or a protein encapsidated with the phage genome, a similar protein was also detected by mass spectrometry for phage AR1^22^.

### Genomic analysis of phage SH6

**Figure 2:**
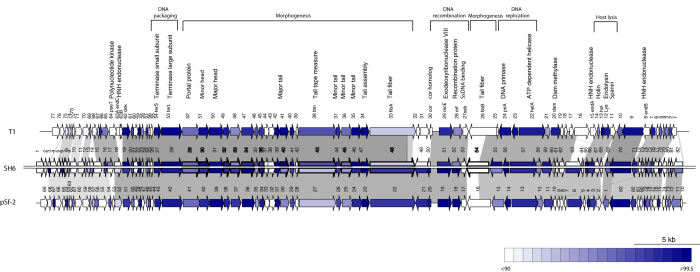
Schematic representation of the genomic organization of phage SH6 compared to phages T1 and pSf-2. Each line represents a different phage genome and each arrow represents a putative protein. The blue gradient represents ORFs whose translated products share from 90 to 99.5% amino acid identity. The upper part of SH6 genome shows identity with phage T1 while the lower part shows identity to phages pSf-2. Conserved genomic regions are connected by grey shading. Arrows with thick outlines and bold numbers represent structural proteins detected by LC-MS/MS.

The genome of phage SH6 is composed of 50,552 bp of double-stranded DNA with 45.8% G+C content, in the same range as phage T1 (48,836 bp; 45.6% G+C)^28^. The SH6 genome is predicted to encode 82 ORFs with only ATG as initiation codon and only 27 ORFs (33%) have assigned functions (Table S1). No tRNA was identified. Phage SH6 is novel but has high nucleotide sequence identity to other phages of the *T1virus* genus including *S. flexneri* phage pSf-2 (89%) and coliphage T1 (83%). Of note, the T1 genome encodes 77 ORFs and is missing homologous sequence to the 3’ region (from ORF78 to ORF82) of phage SH6 (Fig. 2). At the amino acid level, SH6 shares 57 and 48 ORFs with phages pSf-2 and T1, respectively, with more than 90% identity. Of these, SH6 has 6 ORFs with 100% identity to T1 ORFs (ORF46/Minor tail, ORF47/Tail assembly, ORF65/Holin and ORF22, ORF37, ORF39 of unknown functions) and 2 ORFs with 100% identity to phage pSf-2 ORFs (two putative proteins (ORF12, ORF56) of unknown functions). Despite the overall genome conservation between SH6, pSf-2 and T1, diversity occurred upstream of the DNA packaging module. In addition, phage SH6, like pSf-2, is missing the three homing HNH endonucleases dispersed throughout the genome of phage T1. Additional differences between T1 and SH6/pSf-2 were found in the central regions of their genomes: ORF49^SH6^ and ORF49^pSf-2^ share no homology with their T1 counterparts (ORF31 and ORF32) situated in the same genome location but in the opposite orientation. Interestingly, ORF50^SH6^ has 45% identity to the homolog ORF30cor^T1^, a receptor-blocking lipoprotein excluding FhuA-dependent phages^29^. This phage-encoded mechanism protect phage progeny from inactivation by cell wall particles when released from lysed cells^30^.

### Module analysis of phage SH6

The phage SH6 genome can be divided into at least four functional modules, including DNA packaging, phage structure, DNA recombination/replication, and host lysis. The two subunits of the SH6 terminase (ORF27 and ORF28) share 99% aa identity with the phage T1 terminase subunits, suggesting a common packaging process. The terminase complex of phage T1, comprised one small and one large subunit, recognizes a *pac* site to catalyze the headful DNA packaging into preassembled empty capsids using its endonuclease and ATPase activities. This headful mechanism results in terminal redundancy and circular permutation^15^,^28^.

ORF29^SH6^ to ORF48^SH6^ appears to be proteins involved in capsid and tail morphogenesis. In this region, phage SH6 ORFs share more than 95% identity with 79% of T1 ORFs and 68% of pSf-2 ORFs, including the portal protein, the minor and major capsid proteins, the minor and major tail proteins, the tail tape measure protein and the tail assembly protein. This is consistent with the conserved morphology observed between phages of the *T1virus* genus.

Phage SH6 host recognition proteins include two tail fiber proteins, like T1 phages^28^. ORF48^SH6^ is a tail fiber protein of 1,149 amino acids that shares 98% and 92% identity with the homologous ORFs of phages pSf-2 and T1, respectively. Phage SH6 was able to infect the T1 host strain, *E. coli* B11303. However, ORF54^SH6^ appears to be a second 559 aa tail fiber protein, which is the most striking difference between phage SH6 and previously characterized t1viruses. There are few identities between the N-terminal domain of ORF54^SH6^ and the tail fiber proteins of *Shigella* phages Shfl1 (49%) and pSf-2 (45%) as well as with the one of the coliphage T1 (34%). In fact, the C-terminal portion shares identity with the tail tip proteins of *E. coli* phages SSL2009a (50%) and JL1, classified within the *Hk578virus* genus^31^,^32^. It contains a conserved 51 aa peptidase_S74 domain with 94% identity to those two tail tip proteins. Peptidase_S74 is a chaperone of endosialidase that acts as a tailspike protein responsible for host polysialic acid capsule recognition, binding and degrading activity^33^. It seems that ORF54^SH6^ is a combination of the tail fiber proteins of a *T1virus* and a *Hk578virus*, which likely explain the expanded host range of phage SH6 compared to pSf-2, which reportedly could infect only some strains of *S. flexneri*^34^.

Three ORFs constitute the recombination module of phage SH6. ORF51^SH6^, ORF52^SH6^ and ORF53^SH6^ share 98%, 95% and 90% aa identity with ORF29recE^T1^ (exodeoxyribonuclease VIII), ORF28erf^T1^ (recombinase) and ORF27ssb^T1^ (single stranded DNA binding protein), respectively. Two ORFs are within the replication module of SH6, the DNA primase ORF55 and the ATP-dependent helicase ORF57, with high identity (99%) to ORF24priA and ORF22helA of phage T1.

The genome of SH6 contains three closely linked ORFs coding for a holin, an endolysin and a spanin, three proteins that act successively at the end of the latent period for phages release. First, ORF65^SH6^ is 100% identical to ORF13hol^T1^, which encodes a small holin protein (71 aa) and creates pores in the cytoplasmic membrane. Second, ORF66^SH6^ is 99% identical to ORF14Lys^T1^, which encodes the endolysin protein that cleaves β-1,4-linkages in the peptidoglycan of bacterial cell walls^28^. Finally, ORF67^SH6^ has 78% identity to spanin protein ORF11^T1^ which spans the periplasm, making a physical connection between the inner and outer membranes^35^.

### Phylogeny of phage SH6

**Figure 3:**
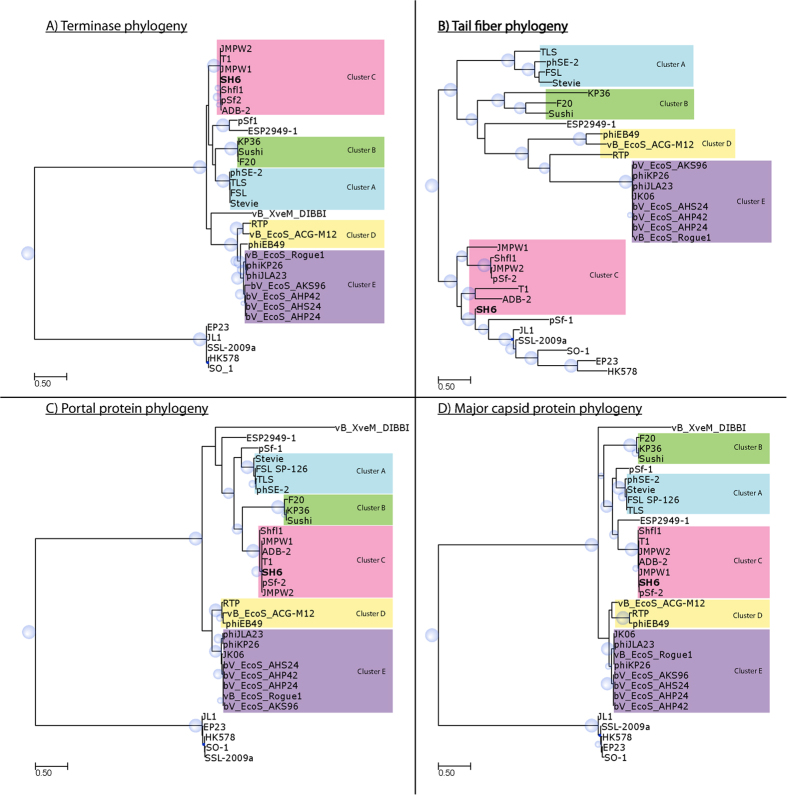
Evolutionary relationships between SH6, the 32 known phages of the *T1virus* genus and the 5 known phages of the *Hk578virus* genus according to four proteins: (**A**) Large subunit of terminase, (**B**) Tail fiber, (**C**) Portal protein and (**D**) Major capsid.

Phylogenetic trees were constructed to further investigate the relatedness of phage SH6 to the 32 known phages of the *T1virus* genus and 5 phages of the *Hk578virus* genus. To associate the newly isolated phage SH6 to one of the previously established clusters^36^, we examined the common features of the same three proteins, including the large terminase (Fig. 3A), the portal protein (Fig. 3C) and the major capsid (Fig. 3D). The phylogenetic trees of each of these three proteins showed that phage SH6 is in the same branch as phages T1, pSf-2, Shfl1, JMPW1, JMPW2 and ADB-2, indicating that these phages belong to cluster C of the *T1virus* genus. Interestingly, examination of a fourth protein, the second tail fiber (ORF54^SH6^), revealed that cluster C was more closely related to phages of the *Hk578virus* genus and phage pSf-1 than to the other phages of the *T1virus* genus (Fig. 3B). It seems that an acquisition of tail fiber protein happened during the evolution of this cluster of *T1virus* genus and the *Hk578virus* genus.

### Genomic analysis of phage SH7

**Figure 4:**
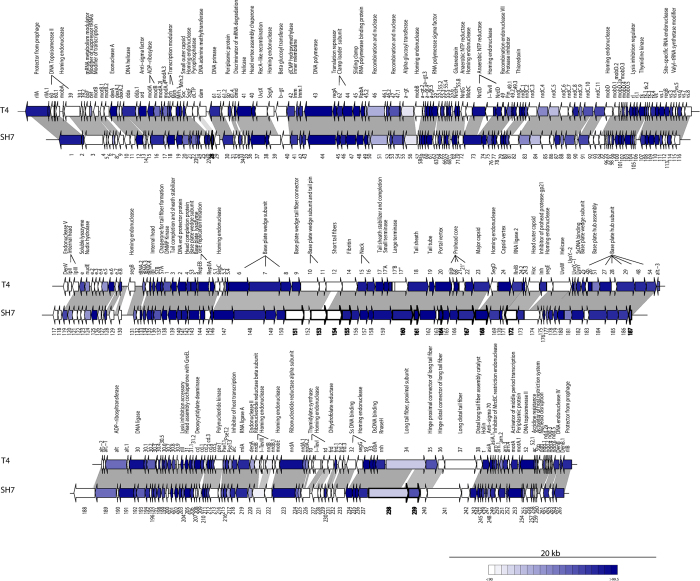
Schematic representation of the genomic organization of phage SH7 compared to phage T4. Each ORF is represented by an arrow. The blue gradient represents ORFs whose translated products share from 90 to 99.5% amino acid identity. Conserved genomic regions are connected by grey shading. Arrows with thick outlines and bold numbers represent structural proteins detected by LC-MS/MS.

The SH7 genome is composed of 164,870 bp of double stranded DNA with a surprisingly low G+C content of 35.5%, as its *Shigella* host has about 50–51% G+C^37^. This low G+C content has been suggested to be advantageous for phage DNA transcription and replication by facilitating its recognition by specific host factors^38^. Phage SH7 genome encodes for 265 putative ORFs (Table S2). The best matches for most of these ORFs are to phages proteins belonging to the T4 superfamily. Of the matches, 137 (52%) could be annotated with a putative function. Comparative genomic analyses with phage T4 revealed that SH7 genes maintain a conserved gene orientation (Fig. 4).

In phage T4, 62 ORFs were previously predicted to be essential, including proteins involved in nucleotide metabolism, transcription regulation and virion structure and assembly^38^. Homologs of all of these essential ORFs were found in the genome of phage SH7 with high identities, including 10 ORFs with 100% identity to phage T4 (ORF74, ORF138, ORF157, ORF158, ORF161, ORF164, ORF180, ORF202, ORF235 and ORF244). A difference was noted in the DNA topoisomerase subunit, which is encoded by a single gene (ORF3) for phage SH7 instead of two genes (gp60^T4^ and gp39^T4^) in T4. However, ORF3^SH7^ shares homology with both T4 genes, suggesting that they have bound or dissociated during the evolutionary process.

Noteworthy, SH7 genome is missing homologs to 14 of the 15 mobile elements encoded by phage T4, including three group I introns (I-Tev (I-III)) and 12 freestanding homing endonucleases genes (7 *seg* (A-G) and 5 *mob* (A-E))^39^. The SH7 genome contains only one homing endonuclease product equivalent to *mob*E^T4^ (62% aa identity), located between the genes coding for the beta and the alpha subunits of the aerobic ribonucleotide reductase. Previous studies also revealed a limited number of such mobile elements for other t4viruses such as coliphages AR1 and RB49^20^,^22^. On the other hand, 11 tRNA genes were identified in the SH7 genome with the following specificities: Arg (UCU), His (GUG), Asn (GUU), Tyr (GUA), Met (CAU), Thr (UGU), Ser (UGA), Pro (UGG), Gly (UCC), Leu (UAA) and Gln (UUG). The number of tRNAs varies considerably among the genomes of phages of the *T4virus* genus^21^. The tRNAs are not essential but are presumably required to ensure optimum progeny size^40^.

Another striking difference between phages T4 and SH7 lies in their tail fiber proteins. Phages of the T4 family recognize their cellular receptor (outer membrane protein C or LPS) using the C-terminus of the distal long tail fiber protein encoded by gp37^T4^-like protein^41^,^42^. The distal long tail fiber protein ORF241^SH7^differs from gp37^T4^, sharing only 37% identity, mostly located at the N-terminus of this protein. In phage T4, the N-terminal region of gp37 is involved in the interaction with its neighboring gp36^T4^ and is highly conserved among the T-even phages^43^. Interestingly, ORF241^SH7^ is 95% identical to the gp37-like protein of phage AR1, most likely explaining the ability of phage SH7 to infect strains of *E. coli* O157:H7 as AR1 is a typing phage for this serotype^22^.

ORF242^SH7^ is likely the tail fiber adhesin implicated in the dimerization of the distal long tail fiber but does not share any homology with gp38^T4^. On the other hand, it is 95% identical to the gp38-like protein of phage wV7, another typing phage of *E. coli* O157:H7^41^. The short tail fibers are encoded by a single gene, gp12^T4^, and their role is to extend and bind irreversibly to the core region of the host LPS^44^. ORF153^SH7^ shares 64% identity with gp12^T4^ and is 99% identical to the short tail fiber proteins of phages e11/12 and AR1.

### Similarity matrix of phage SH7

**Figure 5:**
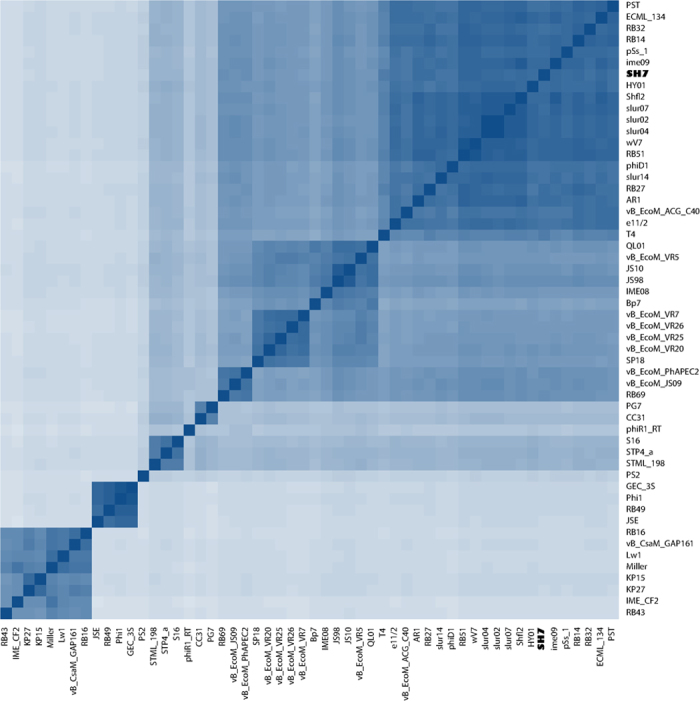
Similarity matrix of 55 phages belonging to the *T4virus* genus available in public databases. The heatmap is generated based on the number of proteins shared by phages. Deeper shade of blue indicates a closer relationship.

Phylogenic analysis of the whole proteome of phage SH7 with the other 54 phages of the *T4virus* genus available in public databases demonstrated that phages within each cluster shared close relatedness at the amino acid level (Fig. 5). This result at the protein level is consistent with the whole genome nucleotide dot blot of the 37 t4viruses^45^. According to the ten clusters defined previously, phage SH7 appears in the same cluster as the prototypic phage T4 (cluster A). This cluster also includes phages PST, ECML_134, RB32, RB14, pSs-1, ime09, HY01, Shfl2, slur07, slur02, slur04, wV7, RB51, phiD1, slur14, RB27, AR1, vB_EcoM_ACG_C40 and e11/12.

## Discussion

In this study, we isolated and characterized two virulent phages, SH6 and SH7, from sewage in Tunisia using a strain of *S. flexneri* as host bacterium. Morphological and genomic analyses revealed that phage SH6 belongs to the *Siphoviridae* family and the *T1virus* genus, while phage SH7 belongs to the *Myoviridae* family and the *T4virus* genus.

Both isolated phages have broad host ranges. Other studies have shown that sequential or simultaneous addition of multiple hosts should be used to isolate phages with broader host range. However with these methods the host range of these phages seems limited to the bacterial species used during their isolation^16^,^46^. The current study indicates that it is possible to isolate such phages using a single host. Phage SH6 infected its host strain *S. flexneri* and 8 strains of *E. coli*. Phage SH7 lysed the same strains as phage SH6 but also strains of *E. coli* O157:H7 (16), *S.* Paratyphi (1), and *S. dysenteriae* (1).

Phages SH6 and SH7 owe their extended host range to the acquisition of new tail fiber genes. Phage SH6 is closely related to phages pSf-2 and T1 infecting, respectively, *S. flexneri* and *E. coli*. The major difference between SH6 and his closely related phages lies in the C-terminal portion of their second tail fiber protein (ORF54^SH6^) which shares similarity with coliphages SSL-2009a and JL1, classified in the *Hk578virus* genus^31^,^32^. On the other hand, phage SH7 is closely related to coliphage T4 but has a wider host range^47^. The major differences between phages SH7 and T4 are in their tail fiber proteins (gp37, gp38 and gp12), which have more similarity to coliphages AR1 and wV7. Since phages AR1 and wV7 are typing phages of *E. coli* O157:H7^22^,^41^, this could explain the ability of phage SH7 to infect this serotype. However, phage AR1 is very specific to *E. coli* O157:H7 and cannot lyse strains of *E. coli* K-12^47^.

The relative lack of specificity of phage SH7 is interesting from a therapeutic perspective. This phage can be amplified on a nonpathogenic host *E. coli* K-12 strain instead of the pathogenic strains of *E. coli* O157:H7, perhaps contributing to an easier approval of a biocontrol product based on this phage^48^.

It has been shown previously that acquisition of new tail fiber genes could be due to recombination between divergent relatives^49^, yet these phages are geographically dispersed and from diverse habitats. Phage AR1 was isolated in 1990 from cow stools in the US^47^, JL1 from sewage in China a few years ago^32^, pSf-2 from sewage in Korea^34^, while SH6 and SH7 were isolated from sewage in Tunisia in 2014. These observations also clearly indicate that related phages are spread around the world, while likely ongoing adaptation to local host strains.

It is also valuable to consider the conditions under which phages can control bacterial populations. For this reason, we investigated the rate and time at which a new phage population can increase, as well as its susceptibility to variable pHs. For phage SH6, the latent period was relatively short (16 min) and the burst size about 103 ± 16 PFU/infected cell. These values are consistent with the range observed for phage T1 (13 min, 100 PFU/infected cell^50^,^51^) but phage SH6 appears more optimized than phage pSf-2 (30 min, 16 PFU/infected cell^34^). The phage SH7 latent period and burst size (23 min, 26 ± 5 PFU/infected cell) are similar to those observed for phages AR1 (20–25 min, 34 PFU/infected cell^47^) and T4 (18–37 min, 12–152 PFU/infected cell^52^). The relative ease of their isolation and their short lytic cycle suggests that these phages were in an environment where new host cells are constantly provided as otherwise they would deplete local resources and possibly go extinct.

Phage SH6 was sensitive to acidic conditions, like phage T1^53^, but stable from pH 5 to 11. On the other hand, phage SH7 was very stable from pH 3 to 11 while phage T4 is sensitive to acidic conditions under pH 5^54^.

While it remains to be seen if these phages can be used for biosanitation purposes or even to treat infectious diseases, these above properties (broader host range, rapid lytic cycle and stability at various pH) will have to be taken into accounts depending of the applications. For example in the case of phage oral application, stomach acid may negatively affect the infectivity of phage SH6. As such, it may require some protection during his passage through the gastrointestinal tract, perhaps through microencapsulation in alginate-chitosan^55^. On the other hand, both phages appear to be highly stable in slightly acidic, neutral, as well as basic solutions or environments.

One of the main limitations to the use of lytic phages for biocontrol or therapeutic purposes is related to the selection of strains resistant to phages. Since phages SH6 and SH7 are highly effective and belong to different phage families, their use in combination may reduce the risk of the rapid emergence of phage resistant strains in the environment. Finally, the newly characterized phages SH6 and SH7 expand our knowledge about the diversity and evolution of enterophages in the environment.

## Materials and Methods

### Bacterial strain, phage isolation and growth conditions

One bacterial isolate was previously obtained by plating a wastewater sample retrieved from the wadi of Megrine in Tunisia on *Salmonella-Shigella* agar (Biokar) and incubating for 24 h at 37 °C. The bacterial species of the isolate was identified using 16S rRNA sequencing and an API 20 E strip test (BioMérieux). This strain was also genotyped using the multi-locus sequence type (MLST) of seven housekeeping genes (*adk, fumC, gyrB, icd, mdh, purA* and *recA*) as described previously^56^. Its allelic profile and sequence type (ST) were determined using the *E. coli* MLST database (http://mlst.warwick.ac.uk/mlst/dbs/Ecoli/dbs/Ecoli). This strain was used as a host for plaque purification, propagation and titration of the phage stocks. The two phages described here were isolated from a water sample retrieved from the wadi of Chotrana in Tunisia as described previously^57^. Phages and hosts are available at the Félix d’Hérelle Reference Center for Bacterial Viruses of the Université Laval (Québec, Canada; www.phage.ulaval.ca) under the following names: phages SH6 (HER521), and SH7 (HER522) as well as *S. flexneri* SF1 (HER1521).

### Microbiological assays

The host range was tested by adding 10 μl of serial dilutions (10^0^ to 10^−6^) of a phage suspension on a lawn of each bacterial strain mixed with 3 ml of BHI containing 0.75% (w/v) agar. Plaque formation was observed after overnight incubation at 37 °C, indicating a complete lytic cycle on the host. For host range analysis, 35 bacterial strains obtained from the Félix d’Hérelle collection were tested (Table 1). The susceptibility of the two phages to different pHs was determined by incubating them in BHI broth adjusted to pH 2 to 11 for one hour as described previously^57^. The one-step growth curve assay was carried out in triplicate. Briefly, phages were mixed with 2 ml of a mid-exponential phase culture of *Shigella flexneri* (optical density at 600 nm of 0.8) with a starting multiplicity of infection (MOI) of 0.05. Phages were allowed to adsorb for 2 min at 37 °C, then the mixture was centrifuged for 30 sec at 16,000x g. The pellet was resuspended in 10 ml of BHI broth. This suspension was incubated at 37 °C and phages were titered every 3 min for a total of 40 min. Plates were incubated for 18–24 h at 37 °C. The burst size was calculated by subtracting the initial titer from the final titer then dividing by the initial titer. The latent phase correspond to the middle of the exponential phase of the curve^58^.

### Electron microscopy

Phages were prepared and observed as described elsewhere^59^. Capsid sizes and tail lengths were determined by measuring at least 10 phage specimens stained with uranyl acetate (2%).

### Structural proteins examination

One liter of phage lysate was concentrated with 10% polyethylene glycol (PEG) 8000 and 29.2 g of sodium chloride (Laboratoire Mat), and purified on a discontinuous CsCl (Fisher Scientific) gradient followed by a continuous CsCl gradient as described previously^60^,^61^. Structural phage proteins were detected directly from the purified phage preparation by liquid chromatography/tandem mass spectrometry (LC-MS/MS) at the Plateforme Protéomique, Centre de Génomique de Québec (Université Laval). A custom database was generated using the putative predicted proteins from the sequences of the phage genomes and compared with the peptides identified. These results were analyzed with Scaffold Proteome software version 4.4.5.

### Genome sequencing and bioinformatics analyses

Phage DNA was extracted from 500 ml of lysate using a Plasmid maxi kit (Qiagen) with modifications^62^. Genome sequencing was performed using the Nextera XT DNA library preparation kit (Illumina) according to the manufacturer’s instructions. The libraries were sequenced using a MiSeq reagent kit v2 (Illumina; 500 cycles) on a MiSeq system. *De novo* assembly was performed with Ray assembler version 2.2.0 using *k*-mer sizes of 31 and 51 and the mean coverage depths for each single phage contig was 5549 and 1369 for SH6 and SH7, respectively. Coverage was calculated with Samtools (http://samtools.sourceforge.net). The open reading frames (ORFs) were identified using NCBI ORF finder (http://www.ncbi.nlm.nih.gov/gorf/orfig.cgi) 63 and GeneMark.hmm prokaryotic^64^ (http://exon.gatech.edu/GeneMark/gmhmmp.cgi). ORFs were analyzed using BioEdit 7.2.0 and validated if they contained at least 30 amino acids (aa), had an initiation codon (AUG, UUG or GUG) and were preceded by a Shine-Dalgarno sequence. BLASTp analysis against the NCBI protein database was used to predict the function of the ORFs. Hits were considered when the E-value was lower than 10^−3^. The percent identity between proteins was calculated by dividing the number of identical residues by the size of the smallest protein. Theoretical molecular weight (MW) and isoelectric point (pI) of the proteins were obtained using the compute pI/MW available on the ExPASy Web page (http://web.expasy.org/compute_pi/). The examination of tRNA genes was conducted with ARAGORN^65^ and tRNAscan-SE v.1.21^66^.

### Phylogeny of phage SH6

The amino acid sequences of the terminase, the second tail fiber protein (fibB^T1^), the neck passage structure and the major capsid protein from a set of phages of the *T1virus* and *Hk578virus* genus were aligned using MAFFT^67^. Since the terminase of phage JK06 is truncated, it was not included in the phylogenetic analysis. Similarly, no orthologous protein to the second tail fiber was found in the genome of phage vB_XveM_DIBBI, so it was not included in the analysis. The best amino-acid substitution model was determined using ProtTest3.4^68^ and was implemented in PhyML 3.0 to calculate the best tree. The Shimodaira-Hasegawa-like procedure was used to determine the branch support values^69^. Finally, Newick utility package^70^ and ETE3.0.0b35^71^ were used to render the trees. All trees were midpoint rooted.

### Similarity matrix of phage SH7

All protein sequences from selected *T4virus* genomes were extracted. The proteins were grouped in orthologous clusters using CogSoft^72^ with an E-value lower than 10^−3^ requiring that the alignment of orthologous proteins span at least 75% of the protein lengths. The clusters were then parsed with in-house Python scripts to obtain a protein presence/absence binary matrix where each column represents a cluster. The rows were populated with the phage genomes. When the phage had a protein that belonged to cluster x, the value 1 was given to the corresponding column/cluster, otherwise the value 0 was given. The similarity matrix was generated in R^73^ with the function *dist* with the binary method to calculate the distance. The heatmap was generated with the gplots package^74^ in R. The order of the rows and columns was manually adjusted when needed.

### Nucleotide sequence accession numbers

The annotated phage genomic sequences were deposited in GenBank under the accession numbers KX828710 (SH6) and KX828711 (SH7).

## Additional Information

**How to cite this article**: Hamdi, S. *et al*. Characterization of two polyvalent phages infecting *Enterobacteriaceae. Sci. Rep.* **7**, 40349; doi: 10.1038/srep40349 (2017).

**Publisher's note:** Springer Nature remains neutral with regard to jurisdictional claims in published maps and institutional affiliations.

## Supplementary Material

Supplementary Information

## Figures and Tables

**Table 1:** Host range of phages SH6 and SH7.

Bacterial species	HER number	SH6	SH7
***Shigella flexneri***			
SF1	1521	+	+
***Escherichia coli B***			
B 11303	1024	+	+
***Escherichia coli*****K-12**			
K12 (λ) Lederberg	1040	+	+
K12 S Lederberg	1144	+	+
K12 S	1037	+	+
K12 C600 (H-19J)	1271	+	+
K12 C600	1275	+	+
K12 C600 (933-J)	1299	+	+
K12 MC 4100	1366	+	+
***Escherichia coli*****O157:H7 serotype**			
C-8299-83	1255	+/−	+
E318	1256	+/−	+
A7793-B1	1257	+/−	+
C8300-83	1258	+/−	+
C7685-84	1259	+/−	+
CL40	1260	+/−	+
C7111-85	1261	+/−	+
B1190-1	1262	+/−	+
B1328-C10	1263	+/−	+
A8188-B3	1264	+/−	+
C7420-85	1265	+/−	+
3283	1266	+/−	+
C7140-85	1267	+/−	+
5896	1268	+/−	+
C-7142-85	1269	+/−	+
C-91-84	1270	+/−	+
***Salmonella*****Paratyphi**			
B type 1	1045	−	+
***Salmonella*****Typhi**			
ViA subtype Tananarive	1038	−	−
Y6R	1043	−	−
***Shigella dysenteriae***			
aSH	1031	−	−
SH (P2)	1020	−	+
***Citrobacter freundii***			
CF3	1518	−	−
CF4	−	−	
CF5	1516	−	−
CF7		−	−
CF8		−	−

(+) Infect; (−) Do not infect; (+/−) Lysis zone at 10^0^ and 10^−1^, likely lysis from without.

**Table 2:** Identified peptides for phages SH6 and SH7 and their predicted functions.

Phage	ORF	Start	Stop	Strand	Predicted function	M.W. (kDa)	Exclusive unique peptide	Sequence coverage (%)
SH6	29	11227	12561	+	Portal protein	50	10	25
	30	12551	13312	+	Minor capsid	29	4	14
	32	14439	14915	+	Unknown function	17	7	55
	33	14978	15745	+	Unknown function	26	5	23
	34	15838	16797	+	Unknown function	35	10	45
	36	17179	17589	+	Unknown function	15	2	16
	40	18787	19455	+	Major tail	24	6	32
	43	20242	23115	+	Tail tape measure	104	28	33
	45	23521	24333	+	Minor tail	30	4	17
	48	25738	29187	+	Tail fiber	127	18	18
	54	34901	33222	−	Tail fiber	60	17	42
SH7	28	15051	14806	−	Small outer capsid	9	2	31
	151	83309	85114	+	Pin	66	4	6
	153	85770	87320	+	Short tail fibers	55	5	9
	154	87330	88787	+	Fibritin neck whiskers	53	7	15
	155	88819	89748	+	Neck	35	3	8
	160	93731	95710	+	Tail sheath monomer	71	8	13
	161	95827	96318	+	Tail tube	18	3	17
	164	98233	98658	+	Prohead core	16	2	13
	167	100155	101720	+	Major capsid	56	13	29
	168	101804	103087	+	Head vertex	47	7	18
	172	105833	104703	−	Large head outer capsid	41	3	8
	187	121210	119150	−	ADP-ribosylase	76	2	3
	238	145822	149694	+	Long tail fiber proximal	140	6	4
	239	150881	151537	+	Hinge connector long tail fiber	23	2	8
