# Supplementary material for: Characterization of two polyvalent phages infecting Enterobacteriaceae
Source: Sci Rep. 2017 Jan 16;7:40349. doi: 10.1038/srep40349 (PMC5238451; doi:10.1038/srep40349)
Supplement: Supplementary Information [file srep40349-s1.pdf]

Table S1. Features of the ORFs of phage SH6, predicted functions and best matches with databases

ORF	Strand	Start (pb)	Stop (pb)	Size(aa)	MW(kDa)	pI	SD sequence(GAGAA) a	Predicted function	Blast (extent, size aligned protein (aa)) b	% aa identity	E Value	Accession number
1	+	6	581	191	17.6	5.7	GACAA**ATG**		pSf2_069 [*Shigella* phage pSf-2] (44/171; 171)	26%	5,00E-06	YP_009113007.1
2	+	584	823	79	9.2	9.3	AAGTAtt**ATG**		B508_00060 [*Escherichia* phage ADB-2] (79/79; 79)	100%	5,00E-50	YP_007112677.1
3	+	895	1089	64	7.3	5.1	ACGAAg**ATG**		Shfl1p02 [*Shigella* phage Shfl1] (64/64; 64)	100%	1,00E-38	YP_004414821.1
4	+	1092	1559	155	18.0	5.7	GGCAAtc**ATG**		Shfl1p03 [*Shigella* phage Shfl1] (143/155; 155)	92%	2,00E-101	YP_004414822.1
5	+	1638	1868	76	8.7	4.8	GAGGAttcacc**ATG**		Shfl1p05 [*Shigella* phage Shfl1] (75/76; 76)	99%	2,00E-47	YP_004414824.1
6	+	1875	2102	75	8.6	9.7	AATAAtc**ATG**		pSf2_064 [*Shigella* phage pSf-2] (74/75; 75)	99%	2,00E-48	YP_009113002.1
7	+	2099	2209	36	4.0	5.2	GAGGTACTAAA**ATG**		pSf2_063 [*Shigella* phage pSf-2] (31/36; 36)	86%	2,00E-13	YP_009113001.1
8	+	2122	2379	85	9.4	8.7	TAGCGct**ATG**		pSf2_062 [*Shigella* phage pSf-2] (53/57; 57)	93%	2,00E-27	YP_009113000.1
9	+	2389	2580	63	7.2	4.1	GATTTaaca**ATG**		pSf2_061 [*Shigella* phage pSf-2] (60/63; 63)	95%	4,00E-36	YP_009112999.1
10	+	2652	3140	162	18.1	5.0	GAGGAtata**ATG**		B508_00095 [*Escherichia* phage ADB-2] (147/162; 172)	91%	2,00E-104	YP_007112684.1
11	+	3212	3718	168	19.0	6.5	GAAAGcatc**ATG**		B508_00100 [*Escherichia* phage ADB-2] (141/168; 168)	84%	5,00E-101	YP_007112685.1
12	+	3718	3867	49	5.6	7.8	GAGAAata**ATG**		pSf2_058 [*Shigella* phage pSf-2] (49/49; 49)	100%	1,00E-26	YP_009112996.1
13	+	3948	4154	68	8.0	6.3	GAGGAatcagct**ATG**		Shfl1p14 [*Shigella* phage Shfl1] (65/68; 68)	96%	2,00E-41	YP_004414831.1
14	+	4138	4425	95	11.2	6.3	AAGAAag**ATG**		pSf2_056 [*Shigella* phage pSf-2] (91/95; 95)	96%	1,00E-60	YP_009112994.1
15	+	4425	5060	212	24.0	9.5	TAGACc**ATG**		pSf2_055 [*Shigella* phage pSf-2] (189/212; 212)	89%	5,00E-137	YP_009112993.1
16	+	5209	5427	72	8.0	7.7	AAGGGtgtttt**ATG**		T1p66 [*Enterobacteria* phage T1] (71/72; 72)	98.0%	1,00E-44	YP_003878.1
17	+	5485	5931	148	17.3	8.3	GGGAAcactc**ATG**		T1p65 [*Enterobacteria* phage T1] (141/148; 148)	95%	3,00E-101	YP_003879.1
18	+	6015	6548	177	20.1	5.6	GAATAaatc**ATG**		morphogenetic protein [*Escherichia* phage ADB-2] (176/177; 203)	99%	3,00E-126	YP_007112690.1
19	+	6657	7229	190	21.7	5.3	GATACtgac**ATG**	Kinase	T1p62 [*Enterobacteria* phage T1] (189/190; 190)	99%	4,00E-138	YP_003883.1
20	+	7302	7511	69	7.9	4.0	TCGAA**ATG**		T1p61 [*Enterobacteria* phage T1] (67/69; 69)	97%	8,00E-41	YP_003884.1
21	+	7508	7852	114	13.1	9.3	AAGAAgc**ATG**		Shfl1p23 [*Shigella* phage Shfl1] (103/114; 114)	90%	2,00E-69	YP_004414839.1
22	+	7852	8082	76	8.8	9.2	GGTAAgta**ATG**		T1p59 [*Enterobacteria* phage T1] (76/76; 76)	100%	9,00E-47	YP_003886.1
23	+	8082	8285	67	7.4	5.1	AAtAAtgg**ATG**		T1p58 [*Enterobacteria* phage T1] (63/67; 67)	94%	9,00E-39	YP_003887.1
24	+	8450	8611	53	6.0	9.2	GAGAAttgcata**ATG**		Shfl1p26 [*Shigella* phage Shfl1] (52/53; 53)	99%	9,00E-28	YP_004414842.1
25	+	8592	8777	61	7.0	9.7	AGGAAg**ATG**		B508_00155 [*Escherichia* phage ADB-2] (60/61; 61)	99%	9,00E-34	YP_007112696.1
26	+	8779	9006	75	8.4	9.7	GAGAtttaac**ATG**		Shfl1p28 [*Shigella* phage Shfl1] (65/75; 75)	87%	1,00E-39	YP_004414844.1
27	+	9091	9615	174	20.2	4.8	ACTAA**ATG**	Terminase small subunit	T1p54 terS [*Enterobacteria* phage T1] (173/174; 174)	99%	2,00E-121	YP_003891.1
28	+	9640	11223	527	60.8	6.0	GCGCCcac**ATG**	Terminase large subunit	T1p53terL [*Enterobacteria* phage T1] (525/527; 527)	99%	0.0	YP_003892.1
29	+	11227	12561	444	50.0	4.9	GGTAAtaa**ATG**	Portal protein	T1p52 [*Enterobacteria* phage T1] (425/427; 427)	99%	0.0	YP_003893.1
30	+	12551	13312	253	29.0	9.5	TAGAAg**ATG**	Minor capsid protein	JMPW1_027 [*Escherichia* phage JMPW1] (252/253; 253)	99%	0.0	ALT58231.1
31	+	13315	14427	370	40.1	5.1	GAGAttaaaa**ATG**	Major capsid protein	pSf2_039 [*Shigella* phage pSf-2] (369/370; 370)	99%	0.0	YP_009112977.1
32	+	14439	14915	158	17.0	5.0	GGGAAatatc**ATG**		T1p49 [*Enterobacteria* phage T1] (156/158; 158)	99%	1,00E-109	YP_003896.1
33	+	14978	15745	255	26.5	4.4	GAGAAatcatt**ATG**		B508_00205 [*Escherichia* phage ADB-2] (250/255; 255)	98%	1,00E-180	YP_007112706.1
34	+	15838	16797	319	35.2	5.6	GAGAAgtaatc**ATG**		B508_00210 [*Escherichia* phage ADB-2] (319/319; 319)	100%	0.0	YP_007112707.1
35	+	16847	17134	95	10.6	4.8	GAGAAatcaaa**ATG**		T1p46 [*Enterobacteria* phage T1] (92/95; 95)	97%	6,00E-58	YP_003899.1
36	+	17179	17589	136	15.5	8.6	GTAAAtt**ATG**		B508_00220 [*Escherichia* phage ADB-2] (134/136; 136)	99%	6,00E-95	YP_007112709.1
37	+	17580	17960	126	14.2	5.0	GTGGAgg**ATG**		T1p44 [*Enterobacteria* phage T1] (123/123; 123)	100%	3,00E-82	YP_003901.1
38	+	17860	18396	178	20.1	9.7	GAGATtg**ATG**		B508_00225 [*Escherichia* phage ADB-2] (157/178; 268)	88%	3,00E-110	YP_007112710.1
39	+	18386	18784	132	15.2	8.7	GCAAA**ATG**		T1p42 [*Enterobacteria* phage T1] (132/132; 132)	100%	3,00E-93	YP_003903.1
40	+	18787	19455	240	26.1	4.5	GAAAGcgg**ATG**	Major tail protein	T1p41 [*Enterobacteria* phage T1] (221/222; 222)	99%	6,00E-161	YP_003904.1
41	+	19569	19886	105	12.0	5.6	GAGAAacatc**ATG**		Shfl1p43 [*Shigella* phage Shfl1] (104/105; 105)	99.0%	2,00E-71	YP_004414859.1
42	+	20006	20203	65	7.5	5.2	CTGAA**ATG**	Tape measure chaperone	B508_00250 [*Escherichia* phage ADB-2] (65/65; 65)	100%	3,00E-39	YP_007112715.1
43	+	20242	23115	957	103.8	5.7	GTTAT**ATG**	Tail tape measure protein	tail tape measure protein [*Escherichia* phage ADB-2] (931/957; 957)	97%	0.0	YP_007112716.1
44	+	23118	23471	117	13.0	5.0	GTTAAt**ATG**	Minor tail protein	putative phage minor tail protein [*Escherichia* phage ADB-2] (116/117; 117)	99%	4,00E-81	YP_007112717.1
45	+	23521	24333	270	30.2	6.2	GTAGA**ATG**	Minor tail protein	T1p36 [*Enterobacteria* phage T1] (259/260; 260)	99%	0.0	YP_003909.1
46	+	24330	25064	244	28.3	5.7	AGGAAaaatca**ATG**	Minor tail protein	T1p35[*Enterobacteria* phage T1] (244/244; 244)	100%	0.0	YP_003910.1
47	+	25061	25660	199	20.9	9.0	TTAAAtt**ATG**	Tail assembly protein	T1p34 [*Enterobacteria* phage T1] (199/199; 199)	100%	4,00E-140	YP_003911.1
48	+	25738	29187	1149	127.3	4.8	GTAAAcatc**ATG**	Tail fiber protein	Shfl1p50[*Shigella* phage Shfl1] (1133/1149; 1149)	99%	0.0	YP_004414866.1
49	-	29215	30171	318	34.2	6.6	GGTAAtt**ATG**		Shfl1p51 [*Shigella* phage Shfl1] (314/318; 318)	99%	0.0	YP_004414867.1
50	-	30174	30404	76	8.3	8.9	GAGGAcatacaa**ATG**	Lipoprotein	pSf2_020 [*Shigella* phage pSf-2] (75/76; 76)	99%	3,00E-47	YP_009112958.1
51	+	30930	31994	354	40.1	5.1	GATAAacgca**ATG**	exodeoxyribonuclease VIII	pSf2_019 [*Shigella* phage pSf-2] (353/354; 354)	99%	0.0	YP_009112957.1
52	+	32069	32716	226	55.6	5.1	GTTATa**ATG**	Recombination	T1p28 erf [*Enterobacteria* phage T1] (224/226; 226)	99%	3,00E-165	YP_003917.1
53	+	32763	33188	141	16.2	6.1	GAGAAaaac**ATG**	Single stranded DNA binding	Shfl1p55 [*Shigella* phage Shfl1] (126/137; 137)	91%	2,00E-77	YP_004414871.1
54	-	33222	34901	559	60.1	4.6	GAGTTtatact**ATG**	Tail fiber	AV954_gp28[*Enterobacteria* phage SSL2009a] (268/538; 538)	50%	4,00E-169	YP_002720067.2
55	-	35002	35922	306	34.6	5.9	AAGAAttt**ATG**	Putative DNA primase	T1p24 priA [*Enterobacteria* phage T1] (303/306; 306)	99%	0.0	YP_003921.1
56	-	35999	36451	150	17.2	10.2	GAGAAtcaag**ATG**		pSf2_014 [*Shigella* phage pSf-2] (150/150; 150)	100%	5,00E-106	YP_009112952.1
57	+	36551	38563	670	75.8	6.8	GTGAca**ATG**	ATP-dependent helicase	T1p22 helA [*Enterobacteria* phage T1] (663/670; 672)	99%	0.0	YP_003923.1
58	+	38560	38976	138	15.9	8.6	CCGGA**ATG**		Shfl1p60 [*Shigella* phage Shfl1] (132/138; 138)	96%	3,00E-94	YP_004414876.1
59	+	39044	39757	237	27.0	6.0	GACTAaag**ATG**	DNA methylase	T1p 20 Dam [*Enterobacteria* phage T1] (234/237; 237)	99%	6,00E-165	YP_003925.1
60	+	39853	40005	83	6.6	4.0	GAGAT**ATG**		T1p19 [*Enterobacteria* phage T1] (50/83; 83)	60%	2,00E-23	YP_003926.1
61	+	40057	40281	74	8.6	9.3	GAAACaaa**ATG**		B508_00360 [*Escherichia* phage ADB-2] (69/69; 69)	100%	9,00E-43	YP_007112737.1

62	+	40362	40763	133	15.5	8.4	TATAAagg**ATG**		Shfl1p66 [*Shigella* phage Shfl1] (88/95; 95)	93%	2,00E-59	YP_004414880.1
63	+	40842	41993	383	43.0	6.5	TAAAAt**ATG**		Shfl1p67 [*Shigella* phage Shfl1] (377/383; 383)	98%	0.0	YP_004414881.1
64	+	42057	42233	58	6.5	6.0	TATATc**ATG**		T1p14 [*Enterobacteria* phage T1] (55/58; 58)	95%	6,00E-32	YP_003931.1
65	+	42352	42567	71	7.6	9.3	GAGATt**ATG**	Holin	T1p13 hol [*Enterobacteria* phage T1] (71/71; 71)	100%	4,00E-42	YP_003932.1
66	+	42567	43055	162	18.3	9.5	GAGATa**ATG**	Endolysin	B508_00380 [*Escherichia* phage ADB-2] (161/162; 162)	99%	2,00E-114	YP_007112741.1
67	+	43055	43456	133	14.3	8.4	AGGAAtt**ATG**	Spanin	Shfl1p71 [*Shigella* phage Shfl1] (125/133; 133)	94%	4,00E-77	YP_004414885.1
68	-	43469	43684	71	8.1	4.7	GAAAGcatc**ATG**		B508_00390 [*Escherichia* phage ADB-2] (67/71; 71)	94%	1,00E-41	YP_007112743.1
69	-	43874	44281	135	16.3	9.2	GTTAAtgat**ATG**		T1p10 [*Enterobacteria* phage T1] (130/135; 135)	96%	6,00E-92	YP_003935.1
70	-	44286	45854	522	58.1	6.3	GTTTAttgtg**ATG**		Shfl1p74 [*Shigella* phage Shfl1] (521/522; 522)	99%	0.0	YP_004414887.1
71	-	45931	46347	138	15.8	9.2	GTGAAtgca**ATG**		T1p07 [*Enterobacteria* phage T1] (136/138; 138)	99%	3,00E-93	YP_003938.1
72	-	46428	46637	69	8.1	3.5	GCTAAtta**ATG**		T1p06 [*Enterobacteria* phage T1] (62/69; 69)	90%	4,00E-37	YP_003939.1
73	-	46641	46865	74	8.0	9.9	TAAAA**ATG**		T1p05 [*Enterobacteria* phage T1] (71/74; 74)	96%	2,00E-41	YP_003940.1
74	-	46943	47086	47	5.1	9.5	AAAAA**ATG**		Shfl1p78 [*Shigella* phage Shfl1] (47/47; 47)	100%	3,00E-25	YP_004414891.1
75	-	47083	47403	106	12.0	9.8	GAGGAATA**ATG**		pSf2_078 [*Shigella* phage pSf-2] (102/106; 106)	96%	4,00E-69	YP_009113016.1
76	-	47419	47619	66	7.5	9.2	GAGGCattgt**ATG**		T1p02 [*Enterobacteria* phage T1] (64/66; 66)	97%	7,00E-38	YP_003943.1
77	-	47612	47983	123	13.7	5.1	GGTAATTAT**ATG**		Shfl1p81 [*Shigella* phage Shfl1] (115/123; 123)	93%	6,00E-79	YP_004414894.1
78	-	47988	48218	76	8.8	6.2	GAGAAtcaccat**ATG**		pSf2_075 [*Shigella* phage pSf-2] (75/76; 76)	99%	3,00E-47	YP_009113013.1
79	-	48294	48629	111	13.2	7.7	GGGAA**ATG**		Shfl1p85 [*Shigella* phage Shfl1] (102/111; 111)	93%	6,00E-69	YP_004414897.1
80	-	48712	48897	61	6.9	7.9	AGCAA**ATG**		pSf2_073 [*Shigella* phage pSf-2] (57/61; 61)	93%	5,00E-32	YP_009113011.1
81	-	48913	49098	61	6.8	8.8	AACAAa**ATG**		B508_00040 [*Escherichia* phage ADB-2] (59/61; 61)	99%	4,00E-33	YP_007112675.1
82	-	49159	49863	234	26.1	7.7	GAGTCacg**ATG**	DNA methylase	putative site specific DNA methylase [*Shigella* phage Shfl1] (228/234; 234)	99%	4,00E-170	YP_004414898.1

a start codon is indicated in boldface, Match to SD sequence is indicated by underlining, SD sequence is indicated in uppercase

b The number of identical amino acids/ The total number of amino acids

Table S2. Features of the ORFs of phage SH7, predicted functions of proteins and best matches with databases

ORF	Start (pb)	Stop (pb)	Strand	Size (aa)	MW (kDa)	pI	SD sequence (GAGAA) a	Predicted function	Blast (extent; Size (aa)) b	% aa identity	E Value	Accession number
1	2178	1	-	725	82.8	5.6	GAGGAaatt**ATG**	Membrane-associated protein ; affects host membrane ATPase	RB32ORF001c [*Enterobacteria* phage RB32](732/725; 725)	99%	0.0	YP_802943.1
2	2392	2189	-	67	8.1	6.6	TTGAA**ATG**		RB32ORF002c [*Enterobacteria* phage RB32](67/67; 67)	100%	3,00E-39	YP_802944.1
3	4264	2447	-	605	68.3	7.2	GATAAgtg**ATG**	DNA topoisomerase subunit DNA-dependent ATPase	60plus39 [*Enterobacteria* phage RB32](605/605; 605)	100%	0.0	YP_802945.1
4	4594	4334	-	86	9.3	7.7	GGTAAtt**ATG**		39.1 [*Enterobacteria* phage RB14] (86/86; 86)	100%	1,00E-55	YP_002854340.1
5	4773	4597	-	59	6.7	8.4	GAATAatt**ATG**	FmdB family transcriptional regulator	RB32ORF006c [*Enterobacteria* phage RB32](58/58; 58)	100%	4,00E-34	YP_802948.1
6	5195	4776	-	139	16.4	4.7	AAATTTATTa**ATG**	mRNA metabolism moderator	F413_gp229 [*Enterobacteria* phage ime09](134/139; 139)	96%	2,00E-93	YP_007004389.1
7	5410	5195	-	71	8.4	5.0	GAAATtgta**ATG**	Modifier of suppressor tRNAs	RB14ORF8 [*Enterobacteria* phage RB14](69/71; 71)	97%	1,00E-41	YP_002854344.1
8	5693	5424	-	89	10.2	5.3	GAGAAtaaa**ATG**		e112_011 [*Escherichia* phage e11/2](89/89; 93)	100%	4,00E-58	YP_009030895.1
9	6288	5791	-	165	18.7	9.1	GAGAGataact**ATG**	MotB/modifier of transcription	RB32ORF009c [*Enterobacteria* phage RB32](165/165; 165)	100%	9,00E-116	YP_802951.1
10	6904	6365	-	179	20.2	5.0	GAGATaatt**ATG**	Adenylribosylating enzyme	motB [*Enterobacteria* phage ime09](176/179;179)	98%	2,00E-124	YP_007004394.1
11	7407	6907	-	166	19.9	5.7	GATAAtaaa**ATG**		ACG-C40_0011 [*Enterobacteria* phage vB_EcoM_ACG-C40](162/166; 166)	98%	9,00E-117	YP_006986562.1
12	8154	7471	-	227	26.0	4.9	AGGAAaattta**ATG**	Exonuclease A	dexA [*Enterobacteria* phage T4] (227/227; 227)	100%	4.27225E-117	NP_049629.1
13	8411	8154	-	85	10.1	7.9	TAGCTATCTTT**ATG**		AR1_015 [*Enterobacteria* phage AR1](82/85; 85)	96%	8,00E-52	YP_009167826.1
14	8523	8389	-	44	5.1	3.8	AGTAAgttg**ATG**		DexA.2 [*Enterobacteria* phage T4] (44/44; 81)	100%	2,00E-21	NP_049631.1
15	8881	8621	-	86	9.9	8.8	GAGGCtat**ATG**		ACQ28_gp015[*Yersinia* phage PST](86/86;86)	100%	2,00E-53	YP_009153618.1
16	10207	8888	-	439	50.0	7.6	GGGACtttccg**ATG**	DNA helicase	D862_gp259 [*Enterobacteria* phage vB_EcoM_ACG-C40](439/439; 439)	100%	0.0	YP_006986566.1
17	10515	10204	-	103	12.1	9.5	GATAAt**ATG**		dda.1 [*Enterobacteria* phage RB51] (103/103; 103)	100%	1,00E-67	YP_002853974.1
18	11263	10517	-	248	29.0	9.9	GAGAAaaat**ATG**		[*Escherichia* phage slur04](247/248; 248)	99%	4,00E-176	CUL02133.1
19	11988	11386	-	249	23.4	5.9	TGAGGTAGTTGA**ATG**	RNA polymerase ADP-ribosylase (anti-sigma factor)	RB27_021 [*Enterobacteria* phage RB27](199/200; 200)	99%	2,00E-145	YP_009102226.1
20	12608	11985	-	208	24.3	5.3	GAGGCtatc**ATG**	Adenylribosylating enzyme	AS348_gp118 [*Escherichia* phage slur14](206/207; 207)	99%	2,00E-149	YP_009180793.1
21	12858	12676	-	60	7.0	4.3	AGGAAttaat**ATG**	Adenylribosylating enzyme	[*Enterobacteria* phage RB51](60/60; 60)	100%	8.9476E-146	YP_002853978.1
22	13337	12867	-	157	18.3	6.1	GAAATcac**ATG**		ACG-C40_0021 [*Enterobacteria* phage vB_EcoM_ACG-C40](151/156; 156)	97%	1,00E-106	YP_006986572.1
23	13494	13330	-	55	6.2	5.8	GATAAatc**ATG**		ModA.4 [*Shigella* phage Shfl2](52/54; 54)	96%	2,00E-28	YP_004414923.1
24	13694	13491	-	67	8.1	6.3	AACAA**ATG**	Transcription modulator	Srh [*Enterobacteria* phage T4] (66/67; 67)	99%	3,00E-38	NP_049640.1
25	14154	13669	-	161	18.5	4.5	GAGGAaaatt**ATG**	Transcription modulator under heat shock	F413_gp214 [*Enterobacteria* phage ime09](161/161; 161)	100%	3,00E-110	YP_007004408.1
26	14504	14163	-	113	12.6	3.9	GAGGCtttttgta**ATG**		slur07_00238 [*Escherichia* phage slur07](112/113; 113)	99%	5,00E-75	CUL02483.1
27	14710	14504	-	69	8.2	5.6	GAGAGcact**ATG**	Affects phosphorylation of host	RB14ORF27 [*Enterobacteria* phage RB14](68/68; 68)	100%	2,00E-42	YP_002854363.1
28	15051	14806	-	81	9.2	5.6	GAGAAttac**ATG**	Small outer capsid protein	RB3_030 [*Enterobacteria* phage RB3] (81/81; 81)	98.6%	3,00E-52	YP_009098416.1
29	15653	15135	-	172	20.2	5.2	GAGAAttac**ATG**	dCTP pyrophosphatase	AS348_gp107 [*Escherichia* phage slur14] (171/172; 172)	99%	3,00E-124	YP_009180804.1
30	16950	15922	-	342	39.8	9.3	GAGTAaaa**ATG**	DNA primase DNA helicase	F413_gp207 [*Enterobacteria* phage ime09](341/342; 342)	99%	0.0	YP_007004416.1
31	17117	16953	-	54	5.9	5.4	GAGGTtaat**ATG**		61.1 [*Enterobacteria* phage RB51](54/54; 54)	100%	5,00E-28	YP_002853989.1
32	17475	17119	-	118	13.8	4.8	GAGGTtagt**ATG**		48 [*Shigella* phage pSs-1](117/118; 118)	99%	1,00E-78	YP_009110855.1
33	17781	17488	-	97	11.0	4.6	GAGATtatc**ATG**	Spackle periplasmic protein	BN81_039 [*Yersinia* phage phiD1](96/97; 97)	99%	2,00E-64	YP_009149278.1
34	18091	17837	-	84	9.7	5.8	GAAAAgaaa**ATG**	RNA metabolism moderator	Shfl2p041 [*Shigella* phage Shfl2](84/84; 84)	100%	2,00E-52	YP_004414938.1
35	18397	18155	-	80	9.6	10.3	GATAAt**ATG**		RB3_037 [*Enterobacteria* phage RB3](79/80; 80)	99%	9,00E-50	YP_009098423.1
36	18581	18399	-	60	7.0	5.2	GAGATtgtt**ATG**	Discriminator of mRNA degradation	dmd [*Enterobacteria* phage T4](60/60; 60)	100%	5.82742E-35	NP_049653.1
37	20067	18640	-	475	53.5	5.5	GAGTAagt**GTG**	DNA primase-helicase subunit	ACQ54_gp036 [*Escherichia* phage HY01](473/475; 475)	99%	0.0	YP_009148487.1
38	20421	20077	-	114	13.3	4.9	AAGAAttta**ATG**	Head vertex assembly chaperone	40 [*Enterobacteria* phage T4](114/114; 114)	100%	5,00E-75	NP_049655.1
39	21595	20414	-	393	44.1	5.4	GAAAAaatga**ATG**	RecA-like recombination protein	F412_gp232 [*Escherichia* phage wV7](392/393; 393)	99%	0.0	YP_007004784.1
40	22515	21673	-	281	32.4	7.1	GAGTGgaa**ATG**	Beta-glucosyltransferase	RB14ORF41 [*Enterobacteria* phage RB14](280/280; 280)	100%	0.0	YP_002854377.1
41	23252	22512	-	247	28.6	5.4	GAGGTttaa**ATG**	DCMP hydroxymethylase	ACQ28_gp041 [*Yersinia* phage PST](246/246; 246)	100%	0.0	YP_009153644.1
42	23657	23406	-	83	9.4	9.4	GAAAAg**ATG**	Immunity to superinfection membrane protein	RB32ORF044c [*Enterobacteria* phage RB32](82/83; 83)	99%	1,00E-48	YP_802986.1
43	24045	23665	-	126	14.2	7.5	GAGAAtgaa**ATG**	Membrane protein	RB51ORF047 [*Enterobacteria* phage RB51](126/126; 126)	100%	1,00E-86	YP_002854002.1
44	26924	24228	-	898	103.6	5.9	GAATAtct**ATG**	DNA polymerase	gp43 [*Enterobacteria* phage T4](897/898; 898)	99%	0.0	NP_049662.1
45	27371	27003	-	122	14.6	8.9	GTAAA**ATG**	Translational repressor protein	regA [*Enterobacteria* phage T4](122/122; 122)	100%	6,00E-84	NP_049663.1
46	27936	27373	-	187	21.4	7.7	AAGAGat**ATG**	Clamp-loader small subunit	slur04_00026 [*Escherichia* phage slur04](187/187; 187)	100%	2,00E-133	CUL01898.1
47	28897	27938	-	319	35.8	7.0	GGAAAtt**ATG**	Clamp-loader subunit	HY01_0048 [*Escherichia* phage HY01](319/319; 319)	100%	0.0	YP_009148499.1
48	29635	28949	-	228	24.9	4.9	GAAATtac**ATG**	Sliding clamp of DNA polymerase	45 [*Enterobacteria* phage RB32](228/228; 228)	100%	6,00E-164	YP_802993.1
49	30080	29691	-	129	14.7	6.8	GTGTAtt**ATG**	RNA polymerase binding protein	rpbA [*Enterobacteria* phage T4](129/129; 129)	100%	3,00E-90	NP_049667.1
50	30290	30090	-	66	7.9	5.0	TATAAtctct**ATG**	Recombination endonuclease subunit	45.2 [*Enterobacteria* phage T4](62/62; 62)	100%	2,00E-37	NP_049668.1
51	31973	30333	-	546	61.8	7.1	CAAAAtata**ATG**	Recombination endonuclease subunit	46 [*Escherichia* phage e11/2](546/546; 560)	100%	0.0	YP_009030664.1
52	32275	32012	-	87	10.6	4.9	TAGATa**ATG**		46.1 [*Enterobacteria* phage AR1](85/87; 87)	98%	4,00E-55	YP_009167869.1
53	32462	32199	-	88	10.2	4.2	GGCAAcact**ATG**		46.2 [*Enterobacteria* phage ime09](87/87; 87)	100%	1,00E-55	YP_007004441.1
54	33478	32459	-	339	39.2	4.9	GATAT**ATG**	Recombination endonuclease subunit	slur04_00034 [*Escherichia* phage slur04](338/339; 339)	99%	0.0	CUL01906.1
55	33615	33475	-	46	5.4	4.2	GATAT**ATG**		47.1 [*Enterobacteria* phage T4] (45/46; 46)	98%	4,00E-21	NP_813808.1
56	34857	33655	-	400	46.9	6.1	GAGGAtattaa**ATG**	α-glucosyltransferase	RB51ORF065 [*Enterobacteria* phage RB51](399/400; 400)	99%	0.0	YP_002854018.1
57	35097	34924	-	57	6.7	9.7	GAAAG**ATG**		RB32ORF061c [*Enterobacteria* phage RB32] (55/57; 57)	96%	2,00E-31	YP_803003.1
58	35304	35101	-	67	7.9	9.4	GATATtgattg**ATG**		RB32ORF062c [*Enterobacteria* phage RB32] (67/67; 67)	100%	1,00E-41	YP_803004.1
59	35590	35273	-	105	12.4	8.8	GAGAAataat**ATG**		α-gt.4 [*Enterobacteria* phage T4](105/105; 105)	100%	3,00E-67	NP_049677.1
60	35810	35592	-	72	8.5	4.2	GATTTtttat**ATG**		α-gt.5 [*Enterobacteria* phage T4](72/72; 72)	100%	7,00E-43	NP_049678.1
61	36351	35794	-	185	21.5	5.4	GTGAAa**ATG**	Sigma factor for late transcription	55 [*Escherichia* phage e11/2](185/185; 187)	100%	2,00E-134	YP_009030673.1
62	36699	36430	-	89	10.8	5.6	GATGAtgt**ATG**		RB14ORF65 [*Enterobacteria* phage RB14](89/89; 89)	100%	3,00E-58	YP_002854401.1
63	36917	36696	-	73	8.3	3.7	GCTAAggt**ATG**		55.1 [*Enterobacteria* phage AR1](73/73; 73)	100%	3,00E-43	YP_009167879.1
64	37240	36914	-	108	12.7	9.7	GATAAtgt**ATG**		ECML134_067 [*Escherichia* phage ECML-134](108/108; 108)	100%	7,00E-71	YP_009102542.1
65	37494	37294	-	66	7.7	7.9	GGGAAatttaa**ATG**		55.3 [*Enterobacteria* phage RB32](66/66; 66)	100%	1,00E-40	YP_803010.1
66	37626	37495	-	43	5.1	8.1	GAGGAaaat**ATG**		55.4 [*Enterobacteria* phage T4](43/43; 43)	100%	5,00E-22	NP_049683.1
67	37927	37634	-	98	11.8	9.7	TTAAActcc**ATG**		55.5 [*Enterobacteria* phage T4](96/97; 97)	99%	6,00E-61	NP_049684.1
68	38096	37920	-	58	6.8	7.9	GAGGAtttgat**ATG**		55.6 [*Enterobacteria* phage ime09](58/58; 58)	100%	3,00E-33	YP_007004455.1
69	38563	38255	-	102	11.7	9.2	AGGAAtagtt**GTG**	Glutaredoxin	nrdH [*Enterobacteria* phage T4](101/102; 102)	99%	6,00E-67	NP_049686.1
70	38778	38566	-	70	7.9	9.2	GTAAAt**ATG**		55.8 [*Enterobacteria* phage T4](70/70; 70)	100%	5,00E-40	NP_049687.1

71	38900	38787	-	75	4.5	4.5	GTAAAt**ATG**	Anaerobic nucleotide reductase subunit	RB32ORF074c [*Enterobacteria* phage RB32] (37/37; 37)	100%	2,00E-17	YP_803016.1
72	39363	38893	-	156	18.2	5.9	GTTAAacacca**ATG**	Ribonucleotide reductase of class III activating protein	F413_gp170 [*Enterobacteria* phage ime09](156/156; 156)	100%	1,00E-110	YP_007004459.1
73	41177	39360	-	605	68.0	6.6	GAGTTtaaa**ATG**	Anaerobic NTP reductase large subunit	Shfl2p080 [*Shigella* phage Shfl2](604/605; 605)	99%	0.0	YP_004414977.1
74	41647	41174	-	158	18.1	8.8	GGGTTttatt**ATG**	Packaging and recombination endonuclease VII	49 [*Enterobacteria* phage T4](157/157; 157)	100%	4,00E-111	NP_049692.1
75	42175	41690	-	161	18.8	4.5	GAGATgggac**ATG**	Inhibitor of host Lon protease	pin [*Enterobacteria* phage RB3](159/161; 161)	99%	2,00E-112	YP_009098466.1
76	42314	42159	-	51	6.2	3.9	ACGAGa**ATG**		49.1 [*Enterobacteria* phage T4] (51/51; 51)	100%	8,00E-28	NP_049695.1
77	42619	42299	-	162	12.6	4.5	GTGAAaaa**ATG**	Nucleotide reductase subunit C	49.2 [*Enterobacteria* phage T4](105/106; 106)	99%	8,00E-71	NP_049696.1
78	43020	42631	-	129	14.9	4.8	GAGAAtattttaa**ATG**		RB32ORF082c [*Enterobacteria* phage RB51](71/71; 71)	100%	2,00E-42	YP_002854042.1
79	43280	43017	-	87	10.0	6.7	GAATAat**ATG**	Thioredoxin, glutaredoxin	nrdC [*Enterobacteria* phage T4](87/87; 87)	100%	3,00E-57	NP_049698.1
80	43524	43282	-	80	9.4	7.9	GAGAAgaaa**ATG**		NrdC.1 [*Enterobacteria* phage T4] (80/80; 80)	100%	2,00E-50	NP_049699.1
81	43825	43511	-	104	12.1	6.1	TTGAA**ATG**		RB32ORF085c [*Enterobacteria* phage RB32](104/104; 104)	100%	3,00E-71	YP_803027.1
82	44751	43822	-	310	35.9	9.3	GAGAAgaac**ATG**	Thioredoxin	HY01_0081 [*Escherichia* phage HY01](302/309; 309)	98%	0.0	YP_009148532.1
83	45835	44804	-	343	40.2	6.2	GTGGTata**ATG**		slur03_00199 [*Escherichia* phage slur03](337/343; 343)	98%	0.0	CUL01789.1
84	46885	45863	-	340	39.5	9.2	AGGAAcact**ATG**		ECML134_087 [*Escherichia* phage ECML-134](321/340; 340)	94%	0.0	YP_009102562.1
85	47784	46894	-	296	33.9	9.2	GAGGAaaat**ATG**		nrdC.6 [*Enterobacteria* phage T4T](292/296; 296)	99%	0.0	ADJ39810.1
86	48200	47796	-	134	15.3	5.7	GAGATtatt**ATG**		ACG-C40_0088 [*Enterobacteria* phage vB_EcoM_ACG-C40] (131/134; 134)	98%	2,00E-90	YP_006986641.1
87	48783	48256	-	176	20.8	6.7	GAACAgaaa**ATG**		nrdC.8 [*Enterobacteria* phage RB14](175/175; 175)	100%	3,00E-121	YP_002854428.1
88	49158	48844	-	104	12.4	9.6	GGTAA**ATG**		nrdC.9 [*Enterobacteria* phage RB32](102/104; 104)	98%	5,00E-69	YP_803034.1
89	50216	49248	-	322	36.4	5.0	GAGAAtaaa**ATG**	Thioredoxin	F412_gp179 [*Escherichia* phage wV7](322/322; 322)	100%	0.0	YP_007004837.1
90	50474	50274	-	67	7.7	6.6	GAGTAacaaa**ATG**		g099/BN81_099 [*Yersinia* phage phiD1](66/66; 66)	100%	1,00E-40	YP_009149338.1
91	51606	50593	-	337	39.0	6.3	TATAAggctga**ATG**		nrdC.11 [*Enterobacteria* phage RB32](334/337; 337)	99%	0.0	YP_803037.1
92	52064	51603	-	153	17.9	9.4	GATAAtt**ATG**		RB51ORF103/RB32ORF096c [*Enterobacteria* phage RB51](152/153; 153)	99%	4,00E-105	YP_002854056.1
93	52588	52067	-	173	19.3	4.9	GAGGAaat**ATG**		AR1_101 [*Enterobacteria* phage AR1](173/173; 173)	100%	2,00E-122	YP_009167912.1
94	53140	52595	-	181	21.0	5.2	GAGTAtgta**ATG**		slur07_00168 [*Escherichia* phage slur07](179/181; 181)	99%	1,00E-128	CUL02413.1
95	53244	53140	-	34	4.2	8.1	GAGAAgaga**ATG**		F412_gp172 [*Escherichia* phage wV7](34/34; 34)	100%	2,00E-13	YP_007004844.1
96	53478	53305	-	58	6.7	5.3	TGGAAta**ATG**		mobD.2a [*Enterobacteria* phage RB14](57/57; 57)	100%	8,00E-33	YP_002854439.1
97	53662	53468	-	65	7.6	5.0	GAGTTtaagt**ATG**		mobD.3 [*Enterobacteria* phage RB51](64/64; 64)	100%	6,00E-38	YP_002854061.1
98	53868	53665	-	67	7.6	4.3	TGGAAta**ATG**		MobD.4 [*Yersinia* phage PST](67/67; 67)	100%	1,00E-37	YP_009153706.1
99	54056	53868	-	62	7.2	4.2	GAGAAcatc**ATG**		g110/BN81_110 [*Yersinia* phage phiD1](62/62; 62)	100%	3,00E-36	YP_009149349.1
100	54538	54152	-	128	14.6	5.5	GAGACtga**ATG**		slur04_00078 [*Escherichia* phage slur04](128/128; 128)	100%	2,00E-86	CUL01950.1
101	54828	54535	-	97	11.1	4.9	GAGGCcttt**ATG**	Membrane protein	rI [*Enterobacteria* phage T4](97/97; 97)	100%	8,00E-65	NP_049717.1
102	55053	54841	-	71	8.3	10.2	GGGTAtat**ATG**		g113/BN81_113 [*Yersinia* phage phiD1](70/70; 70)	100%	8,00E-42	YP_009149352.1
103	55677	55096	-	194	21.6	6.2	GACTAag**ATG**	Thymidine kinase	tk [*Enterobacteria* phage RB32](193/193; 193)	100%	2,00E-140	YP_803049.1
104	55867	55679	-	62	7.3	4.1	TATAAattcctt**ATG**		slur07_00159 [*Escherichia* phage slur07](62/62; 62)	100%	2,00E-33	CUL02404.1
105	56049	55864	-	61	7.2	4.5	GAAATtaatat**ATG**		tk.2 [*Enterobacteria* phage RB32](61/61; 61)	100%	8,00E-34	YP_803051.1
106	56219	56046	-	57	6.6	5.2	GTAAAccgaa**ATG**		RB14ORF113/RB32ORF110c [*Enterobacteria* phage RB14](57/57; 57)	97.6%	1,00E-30	YP_002854449.1
107	57095	56628	-	156	17.5	6.3	GAGTActag**ATG**		tk.4 [*Enterobacteria* phage RB51](154/155; 155)	99%	9,00E-110	YP_002854072.1
108	57439	57092	-	115	13.1	9.0	AGTAA**ATG**	valyl-tRNA synthetase modifier	vs [*Enterobacteria* phage T4] (115/115; 115)	100%	6,00E-77	NP_049724.1
109	57977	57432	-	181	20.7	9.7	GGTAAgc**ATG**		vs.1 [*Enterobacteria* phage T4](181/181; 181)	100%	1,00E-128	NP_049725.1
110	58446	57985	-	153	18.0	8.9	GAGAAtaac**ATG**	Site-specific RNA endonuclease	regB [*Enterobacteria* phage T4](152/153; 153)	99%	8,00E-108	NP_049726.1
111	58784	58506	-	93	10.9	5.4	GAAAAatacta**ATG**		RB27_120 [*Enterobacteria* phage RB27](92/92; 92)	100%	6,00E-60	YP_009102325.1
112	59080	58784	-	98	11.4	4.9	TTGAAt**ATG**		vs.4 [*Enterobacteria* phage AR1](95/98; 98)	97%	2,00E-61	YP_009167932.1
113	59264	59043	-	73	8.2	4.1	GAGTCtgta**ATG**		vs.5 [*Enterobacteria* phage RB27](73/73)	100%	6,00E-46	YP_009102327.1
114	59626	59264	-	120	13.8	5.8	GAGGTttat**ATG**	Pyruvate formate-lyase	vs.6 [*Enterobacteria* phage RB51] (120/120; 120)	100%	2,00E-81	YP_002854079.1
115	60014	59634	-	126	15.0	9.3	GTTAA**ATG**		vs.7 [*Enterobacteria* phage T4](124/126; 126)	98%	1,00E-86	CAA28221.1
116	60499	59960	-	179	20.2	8.8	GAGAAgaa**ATG**		slur14_00014 [*Escherichia* phage slur14](178/179; 179)	99%	3,00E-129	YP_009180624.1
117	61113	60640	-	157	17.8	8.9	AGGAAacac**ATG**	Internal head protein II	ipII [*Enterobacteria* phage RB32](157/157; 157)	100%	2,00E-109	YP_803065.1
118	61447	61130	-	105	12.1	9.4	AGGAAacac**ATG**	Internal head protein III	slur04_00097 [*Escherichia* phage slur04] (105/105; 105)	100%	2,00E-70	CUL01969.1
119	62030	61536	-	164	18.6	9.6	GAGGTatt**ATG**	Endolysin	e [*Enterobacteria* phage RB32](164/164; 164)	100%	5,00E-117	YP_803066.1
120	62523	62068	-	151	17.6	5.2	TATCActga**ATG**	Nudix hydrolase	e112_129 [*Escherichia* phage e11/2](147/151; 151)	97%	9,00E-107	YP_009030734.1
121	62993	62505	-	162	19.4	8.4	GAAAAttaa**ATG**	Membrane protein	e.2 [*Enterobacteria* phage RB51](162/162; 162)	97.8%	9,00E-114	YP_002854086.1
122	63352	62990	-	120	14.1	7.7	AGGAAtcacg**ATG**	Membrane protein	e.3 [*Enterobacteria* phage RB3] (119/120; 120)	99%	1,00E-80	YP_009098516.1
123	63726	63334	-	130	15.2	9.6	GAGAAt**ATG**		e.4 [*Enterobacteria* phage RB32] (127/130; 130)	98%	3,00E-85	YP_803069.1
124	64309	63695	-	204	24.1	5.4	GTAAAtagga**ATG**		gp146/RM00_gp144 [*Shigella* phage pSs-1] (201/204; 204)	99%	3,00E-145	YP_009110952.1
125	64944	64351	-	197	22.1	6.1	AGGAAatact**ATG**		gp147/RM00_gp145 [*Shigella* phage pSs-1] (197/197; 197)	100%	9,00E-134	YP_009110953.1
126	65337	65002	-	111	13.0	4.3	GAAAAcatt**ATG**		slur04_00106 [*Escherichia* phage slur04] (110/111; 111)	99%	7,00E-73	CUL01978.1
127	65546	65382	-	54	6.1	4.0	GAGAAataa**ATG**		RB69p145/RB69ORF145c [*Enterobacteria* phage RB69] (53/54; 54)	98%	4,00E-30	NP_861835.1
128	65878	65615	-	87	10.2	4.5	GAGAAcgaa**ATG**		e.8 [*Enterobacteria* phage ime09] (87/87; 109)	100%	4,00E-57	YP_007004513.1
129	66583	66110	-	157	17.2	9.5	AGGAAataag**ATG**		ACQ54_gp126 [*Escherichia* phage HY01] (156/157; 157)	99%	1,00E-106	YP_009148577.1
130	67319	66969	-	116	14.2	6.5	AGGAAaat**ATG**		F413_gp248 [*Enterobacteria* phage ime09] (114/116; 116)	98%	9,00E-79	YP_007004516.1
131	68742	68455	-	95	11.3	4.9	GTTGAta**ATG**		ACQ54_gp129 [*Escherichia* phage HY01] (94/95; 95)	99%	2,00E-61	YP_009148580.1
132	69125	68745	-	126	14.3	4.6	GTGAAtaga**ATG**		F413_gp246 [*Enterobacteria* phage ime09] (126/127; 127)	99%	1,00E-85	YP_007004518.1
133	69312	69127	-	61	6.6	7.8	GAGAAtaaa**ATG**		tRNA.4 [*Enterobacteria* phage T4] (61/61; 61)	100%	9,00E-33	NP_049748.1
134	69676	69389	-	95	10.2	8.9	AAAAA**ATG**	internal head protein	ACQ54_gp132 [*Escherichia* phage HY01] (95/95; 95)	100%	3,00E-59	YP_009148583.1
135	70206	69751	-	151	17.1	5.1	AAGAAta**ATG**		gp57B [*Enterobacteria* phage T4] (151/151; 152)	100%	2,00E-106	NP_049750.1
136	70448	70206	-	80	8.7	4.4	AGGTActata**ATG**	Chaperone for long and short tail fiber formation	Shfl2p143 [*Shigella* phage Shfl2] (80/80; 80)	100%	3,00E-45	YP_004415038.1
137	71161	70448	-	237	26.9	5.3	GAGAAacat**ATG**	Deoxynucleotide monophosphate kinase	slur14_00046 [*Escherichia* phage slur14] (234/237; 237)	99%	5,00E-172	YP_009180646.1
138	71741	71211	-	176	19.7	4.4	GAGGAatat**ATG**	Tail completion and sheath stabilizer protein	3 [*Enterobacteria* phage T4] (176/176; 184)	100%	2,00E-125	AAA50419.1
139	72672	71848	-	274	31.6	10.1	GGCATa**ATG**	DNA end protector protein	2 [*Enterobacteria* phage T4] (273/274; 274)	99%	0.0	NP_049754.1
140	73124	72672	-	150	17.6	9.6	GAGAAtcca**ATG**	Head completion protein	4 [*Enterobacteria* phage RB32] (150/150; 150)	100%	3,00E-105	YP_803090.1
141	73172	73762	+	196	23.0	5.9	GAGGGccc**ATG**	Baseplate wedge subunit	Shfl2p149 [*Shigella* phage Shfl2] (195/196; 196)	99%	1,00E-140	YP_004415043.1
142	73746	75473	+	575	63.1	5.3	GAGAAatctt**ATG**	Baseplate hub subunit and lysozyme	slur07_00109 [*Escherichia* phage slur07] (574/575; 575)	99%	0.0	CUL02363.1

143	73905	73768	-	45	5.5	4.9	GACATcc**ATG**	Replication initiation protein	RepEB oriE [*Enterobacteria* phage T4] (43/45; 45)	96%	7,00E-21	NP_049758.1
144	75006	74872	-	44	5.4	5.3	CTCAT**ATG**	Replication initiation protein	RepEA oriE [*Enterobacteria* phage T4] (35/43; 50)	81%	4,00E-13	NP_049759.1
145	75448	76002	+	184	20.6	4.5	CACAAttg**ATG**		e112_162 [*Escherichia* phage e11/2] (182/184; 184)	99%	6,00E-129	YP_009030759.1
146	76003	76296	+	97	10.2	8.6	CATAA**ATG**		5.4 [*Enterobacteria* phage T4] (97/97; 97)	100%	2,00E-63	NP_049763.1
147	76305	78287	+	660	74.3	4.6	GATTTaaa**ATG**	Baseplate wedge component	6 [*Yersinia* phage PST] (659/660; 660)	99%	0.0	YP_009153751.1
148	78284	81382	+	1033	118.9	5.0	TATATcaca**ATG**	Baseplate wedge component	gp7 [*Enterobacteria* phage AR1] (1025/1032; 1032)	99%	0.0	YP_009167970.1
149	81375	82379	+	334	38.0	4.6	TAAAAtag**ATG**	Baseplate wedge component	ECML134_151 [*Escherichia* phage ECML-134] (333/334; 334)	99%	0.0	YP_009102626.1
150	82443	83309	+	288	31.0	5.0	GAAACcgct**ATG**	Baseplate wedge tail fiber connector	9 [*Enterobacteria* phage T4] (285/288; 288)	99%	0.0	NP_049767.1
151	83309	85114	+	601	66.3	4.5	CTCAAta**ATG**	Baseplate wedge subunit and tail pin	10 [*Escherichia* phage e11/2] (601/601; 601)	100%	0.0	YP_009030765.1
152	85114	85773	+	219	24.1	5.2	TTGCATA**ATG**	Baseplate wedge subunit and tail pin	11 [*Escherichia* phage e11/2] (219/219; 219)	100%	8,00E-159	YP_009030766.1
153	85770	87320	+	516	55.4	5.8	GAGAAtagc**ATG**	Short tail fibers	12 [*Escherichia* phage e11/2] (514/516; 516)	99%	0.0	YP_009030767.1
154	87330	88787	+	486	52.6	4.8	TACAA**ATG**	Fibritin neck whiskers	wac [*Enterobacteria* phage RB27] (475/485; 485)	98%	0.0	YP_009102364.1
155	88819	89748	+	309	34.7	4.9	AAGGAttttaa**ATG**	Neck protein	Shfl2p161 [*Shigella* phage Shfl2](308/309; 309)	99%	0.0	YP_004415055.1
156	89750	90520	+	256	29.6	4.6	GTTAAt**ATG**	Neck protein	14 [*Enterobacteria* phage RB3] (256/256; 256)	100%	0.0	YP_009098546.1
157	90562	91380	+	272	31.6	4.9	GAGAAatc**ATG**	Tail sheath stabilizer and completion	15 [*Enterobacteria* phage T4] (272/272; 272)	100%	0.0	NP_049774.1
158	91389	91883	+	164	18.4	4.6	GAGGTtatt**ATG**	Terminase DNA packaging enzyme small subunit	16 [*Enterobacteria* phage T4] (164/164; 164)	100%	4,00E-116	NP_049775.1
159	91867	93699	+	610	69.8	5.6	GATAA**ATG**	Terminase large subunit nuclease and ATPase	17 [*Enterobacteria* phage RB32] (610/610; 610)	100%	0.0	YP_803107.1
160	93731	95710	+	659	71.3	4.8	GATTAaaa**ATG**	Tail sheath monomer	g171 [*Yersinia* phage phiD1] (656/659; 659)	99%	0.0	CCI89062.1
161	95827	96318	+	163	18.5	4.7	GCTAAt**ATG**	Tail tube monomer	19 [*Enterobacteria* phage T4] (163/163; 163)	100%	4,00E-116	NP_049781.1
162	96402	97976	+	524	61.0	5.4	GAGAAtaca**ATG**	Portal vertex protein of the head	20 [*Enterobacteria* phage RB3] (524/524; 524)	100%	0.0	YP_009098553.1
163	97976	98233	+	85	9.7	3.8	GAGGAttttta**ATG**	Prohead core	gp67 /F413_gp094 [*Enterobacteria* phage ime09] (85/85; 85)	100%	9,00E-45	YP_007004547.1
164	98233	98658	+	141	15.9	10.1	GATGAata**ATG**	Prohead core	68 [*Enterobacteria* phage T4] (141/141; 141)	100%	7,00E-95	NP_049784.1
165	98658	99296	+	212	23.3	5.0	GATTAagcta**ATG**	Prohead core scaffold protein and protease	21 [*Enterobacteria* phage RB51] (212/212; 212)	100%	2,00E-151	YP_002854128.1
166	99327	100136	+	269	29.8	4.6	TACAAa**ATG**	Prohead core scaffold protein	22 [*Escherichia* phage ECML-134] (268/269; 269)	99%	0.0	YP_009102644.1
167	100155	101720	+	521	56.1	5.3	CACAA**ATG**	Precursor of major head subunit	gp187 /RM00_gp185 [*Shigella* phage pSs-1] (521/521; 521)	100%	0.0	YP_009110993.1
168	101804	103087	+	427	47.0	4.7	GCGCA**ATG**	Precursor of head vertex subunit	ACQ54_gp165 [*Escherichia* phage HY01] (427/427; 427)	100%	0.0	AHK11022.1
169	104124	103120	-	334	37.6	5.4	GATTAatt**ATG**	RNA ligase	Shfl2p177 [*Shigella* phage Shfl2] (328/334; 334)	98%	0.0	YP_004415069.1
170	104412	104134	-	92	11.0	5.0	GTGAAaa**ATG**		24.2 [*Enterobacteria* phage T4] (92/92; 92)	100%	9,00E-60	NP_049791.1
171	104614	104399	-	71	8.3	10.5	GATAAc**ATG**		24.3 [*Enterobacteria* phage RB32] (71/71; 71)	100%	1,00E-41	YP_803119.1
172	105833	104703	-	377	40.5	4.6	GATAActt**ATG**	Large capsid outer capsid	hoc [*Enterobacteria* phage RB32] (339/376; 376)	90%	0.0	YP_803120.1
173	106523	105843	-	226	25.6	4.5	GAGGAaatatt**ATG**	Minor capsid protein inhibitor of prohead protease	inh [*Enterobacteria* phage RB27] (225/226; 226)	99%	2,00E-159	YP_009102383.1
174	106574	108085	+	503	57.9	9.3	TAGAC**ATG**	RNA-DNA and DNA- helicase; DNA dependent ATPase	uvsW [*Enterobacteria* phage RB51] (503/503; 503)	100%	0.0	YP_002854137.1
175	108111	108341	+	76	8.8	4.2	GAGAAaaag**ATG**	RNA-DNA and DNA- helicase; DNA dependent ATPase	uvsW [*Escherichia* phage e11/2] (76/76; 80)	100%	4,00E-44	YP_009030789.1
176	108564	108397	-	55	6.1	4.4	GACAAtc**ATG**		uvsY.-2 [*Enterobacteria* phage T4] (55/55; 100)	100%	1,00E-30	NP_049797.1
177	108915	108688	-	75	9.1	4.9	GAAAAta**ATG**		uvsY.-1 [*Escherichia* phage e11/2] (75/75; 75)	100%	6,00E-47	YP_009030792.1
178	109328	108915	-	137	15.8	7.8	GAGAAcaat**ATG**	Repair and ssDNA binding	uvsY [*Enterobacteria* phage RB32] (137/137; 137)	100%	9,00E-93	YP_803126.1
179	109793	109395	-	132	15.1	4.6	GAGTTactata**ATG**	Baseplate wedge subunit	slur04_00168 [*Escherichia* phage slur04] (132/132; 132)	100%	5,00E-89	CUL02029.1
180	110419	109793	-	208	23.9	5.6	GAGCTaaat**ATG**	Baseplate hub subunit	26 [*Enterobacteria* phage T4] (208/208; 208)	100%	2,00E-149	NP_049801.1
181	110470	111219	+	249	29.3	5.8	GAGCAtct**ATG**	Baseplate hub assembly catalyst	51 [*Enterobacteria* phage ime09] (247/249; 249)	99%	8,00E-180	YP_007004565.1
182	111219	112394	+	391	44.4	5.2	AACAAta**ATG**	Baseplate hub subunit	27 [*Enterobacteria* phage RB32] (390/391; 391)	99%	0.0	YP_803130.1
183	112414	112872	+	153	17.3	5.0	CTAAA**ATG**	Baseplate hub distal subunit	28 [*Enterobacteria* phage RB32] (152/152; 177)	100%	5,00E-106	YP_803131.1
184	112869	114641	+	590	64.3	5.1	GATTAtttga**ATG**	Baseplate hub subunit and tail length determinator	Shfl2p194 [*Shigella* phage Shfl2] (581/590; 590)	98%	0.0	YP_004415085.1
185	114650	115744	+	364	39.7	8.9	GAATAat**ATG**	Tail-tube assembly	slur14_00092 [*Escherichia* phage slur14] (361/364; 364)	99%	0.0	YP_009180692.1
186	115744	116709	+	321	34.9	5.1	GACTTttta**ATG**	baseplate tail tube initiator	D862_gp081 [*Enterobacteria* phage vB_EcoM_ACG-C40] (319/321; 321)	99%	0.0	YP_006986749.1
187	117028	116738	-	96	10.7	4.7	CACAAa**ATG**		alt.-3 [*Enterobacteria* phage RB51] (96/96; 96)	100%	3,00E-59	YP_002854150.1
188	119146	117089	-	685	75.9	5.8	GATAAatc**ATG**	RNA polymerase ADP-ribosylase	F412_gp080 [*Escherichia* phage wV7] (679/685; 685)	99%	0.0	YP_007004936.1
189	121210	119150	-	686	76.4	5.7	GAGGAt**ATG**	RNA polymerase ADP-ribosylase	alt [*Enterobacteria* phage AR1] (681/685; 685)	99%	0.0	YP_009168013.1
190	121448	121260	-	62	7.1	4.5	GGTAAggttt**ATG**		Shfl2p200 [*Shigella* phage Shfl2] (62/62; 62)	100%	6,00E-36	YP_004415091.1
191	122908	121445	-	487	55.3	6.1	GATGAaca**ATG**	DNA ligase	30 [*Yersinia* phage PST] (485/487; 487)	99%	0.0	YP_009153798.1
192	123174	122905	-	89	10.8	7.8	GAGCAttcta**ATG**		30.1 [*Enterobacteria* phage LZ9] (89/89; 89)	100%	7,00E-60	CAJ32735.1
193	124013	123174	-	279	32.4	5.9	GAGACtctct**ATG**		30.2 [*Enterobacteria* phage RB32] (275/279; 279)	99%	0.0	YP_803141.1
194	124468	124010	-	152	17.2	9.0	TTGAAa**ATG**		30.3 [*Enterobacteria* phage T6] (150/152; 152)	99%	3,00E-106	CAD30031.1
195	124667	124461	-	68	8.0	5.0	AGGAAttagt**ATG**		30.4 [*Enterobacteria* phage AR1] (67/68; 68)	99%	3,00E-40	YP_009168019.1
196	124861	124664	-	65	7.2	5.2	GAGGTtctg**ATG**		30.5 [*Enterobacteria* phage RB51] (65/65; 65)	100%	3,00E-37	YP_002854159.1
197	125148	124861	-	95	10.8	6.7	GAAAAtact**ATG**		30.6 [*Enterobacteria* phage RB14] (95/95; 95)	100%	1,00E-63	YP_002854539.1
198	125554	125189	-	121	14.2	6.1	GAGAAtaaaa**ATG**		Shfl2p208 [*Shigella* phage Shfl2] (121/121; 121)	100%	5,00E-85	YP_004415099.1
199	125955	125623	-	110	12.9	6.7	GAGAAtaaa**ATG**		30.8 [*Enterobacteria* phage RB51] (109/110; 110)	99%	3,00E-72	YP_002854162.1
200	126284	126066	-	72	8.2	11.3	AGGCA**ATG**		30.9 [*Yersinia* phage PST] (72/72; 72)	100%	7,00E-42	YP_009153807.1
201	126737	126489	-	82	9.3	8.1	GAGAAtttttaa**ATG**	Lysis inhibition accessory protein, rapid lysis phenotype	rIII [*Enterobacteria* phage T4] (82/82; 82)	100%	5,00E-51	NP_049824.1
202	127220	126885	-	111	12.1	5.3	GAAAAacg**ATG**	Head assembly cochaperone with GroEL	31 [*Enterobacteria* phage T4] (111/111; 111)	100%	4,00E-72	NP_049825.1
203	127585	127277	-	102	11.5	8.0	GTAAAtaa**ATG**		31.1 [*Enterobacteria* phage T4] (101/102; 102)	99%	2,00E-65	NP_049826.1
204	127822	127586	-	78	9.3	9.6	GTGAAta**ATG**	Tail fiber	RB27_209 [*Enterobacteria* phage RB27] (77/78; 78)	99%	5,00E-48	YP_009102414.1
205	128403	127822	-	193	21.2	8.0	ATGAA**ATG**	dCMP deaminase	cd [*Enterobacteria* phage RB32] (193/193; 193)	100%	8,00E-140	YP_803153.1
206	128735	128400	-	112	12.7	7.9	TACTAtg**ATG**		RB27_211 [*Enterobacteria* phage RB27] (111/111; 112)	100%	4,00E-74	YP_009102416.1
207	128974	128735	-	80	89.3	5.3	GATCAac**ATG**		cd.2 [*Enterobacteria* phage RB51] (79/79; 79)	100%	6,00E-48	YP_002854170.1
208	129138	128965	-	57	6.5	4.6	GGTATtactgg**TTG**		AR1_225 [*Enterobacteria* phage AR1] (57/57; 176)	100%	3,00E-30	YP_009168036.1
209	129495	129217	-	92	10.8	4.9	GAGGAaattgtt**ATG**		ACQ54_gp205 [*Escherichia* phage HY01] (92/92; 175)	100%	3,00E-59	YP_009148656.1
210	129833	129558	-	92	10.1	4.9	GAGGTaaat**ATG**		Cd.3 [*Enterobacteria* phage T4] (91/91; 91)	100%	3,00E-58	NP_049831.1
211	130036	129836	-	66	7.9	4.4	GAAAActt**ATG**		cd.4 [*Enterobacteria* phage AR1] (66/66; 66)	100%	4,00E-39	YP_009168038.1
212	130256	130029	-	75	8.6	8.7	TTGAA**ATG**		cd.5 [*Enterobacteria* phage AR1] (74/75; 75)	99%	2,00E-46	YP_009168039.1
213	131131	130226	-	301	35.2	8.4	AGGAAttaa**ATG**	Deoxyribonuleotide 3' phosphatase; 5' Polynucleotide kinase	pseT [*Enterobacteria* phage RB27] (286/301; 301)	95%	0.0	YP_009102420.1
214	131430	131128	-	100	11.5	8.5	GAGAAaaataa**ATG**		F412_gp055 [*Escherichia* phage wV7](98/100; 100)	98%	2,00E-63	YP_007004961.1

215	131657	131427	-	76	8.9	7.9	GATAAatgtaa**ATG**		pseT.1 [*Enterobacteria* phage RB32] (75/76; 76)	99%	3,00E-47	YP_803160.1
216	131953	131654	-	99	11.6	8.8	GACCTttctaa**ATG**		pseT.2 [*Enterobacteria* phage RB32] (98/99; 99)	99%	2,00E-65	YP_803161.1
217	132303	131950	-	117	13.0	8.9	TAAAActtt**ATG**	Membrane protein	slur07_00036 [*Escherichia* phage slur07] (114/117; 117)	97%	5,00E-75	CUL02290.1
218	132800	132294	-	168	19.1	6.4	GAGGActtt**ATG**		slur04_00206 [*Escherichia* phage slur04] (167/168; 168)	99%	2,00E-120	CUL02067.1
219	133986	132862	-	374	43.5	4.9	GAGGAtacaca**ATG**	RNA ligase 1 and tail fiber attachment catalyst	rnlA [*Enterobacteria* phage AR1] (372/374; 374)	99%	0.0	YP_009168046.1
220	134449	134039	-	136	15.8	9.2	GCCCAttttatt**ATG**	Endonuclease II	denA [*Enterobacteria* phage RB51] (136/136; 136)	100%	5,00E-94	YP_002854181.1
221	135655	134477	-	392	46.0	5.0	GAAAAatta**ATG**	Ribonucleotide reductase β subunit	F412_gp048 [*Escherichia* phage wV7] (376/392; 392)	96%	0.0	YP_007004968.1
222	136386	135655	-	243	28.0	9.6	GTAAAttgta**ATG**	Homing endonuclease	mobE [*Enterobacteria* phage RB3] (235/243; 243)	97%	1,00E-175	YP_009098612.1
223	138650	136386	-	754	86.0	5.9	GAGGActt**ATG**		nrdA [*Enterobacteria* phage T6] (752/754; 754)	99%	0.0	ABI48936.1
224	138967	138641	-	108	12.4	9.2	AACAAt**ATG**		Shfl2p233 [*Shigella* phage Shfl2] (108/108; 108)	100%	3,00E-74	YP_004415123.1
225	139184	138921	-	87	10.0	5.2	GAGGAtat**ATG**		ECML134_228 [*Escherichia* phage ECML-134] (86/87; 87)	99%	1,00E-55	YP_009102703.1
226	140068	139208	-	286	33.2	6.3	ATGAActa**ATG**	thymidylate synthase	RM00_gp240 [*Shigella* phage pSs-1] (284/286; 286)	99%	0.0	YP_009111048.1
227	140856	140272	-	194	21.9	5.3	GAGGAtttgtgca**ATG**	Dihydrofolate reductase	RM00_gp242 [*Shigella* phage pSs-1] (189/194; 194)	97%	2,00E-137	YP_009111050.1
228	141113	140853	-	86	10.3	3.9	GACCCttttca**ATG**		D862_gp039 [*Enterobacteria* phage vB_EcoM_ACG-C40] (79/81; 81)	98%	5,00E-49	YP_006986792.1
229	141457	141098	-	119	13.3	4.9	GAGAAaaatatt**ATG**		ACQ54_gp227 [*Escherichia* phage HY01] (109/119; 119)	92%	5,00E-75	YP_009148676.1
230	141710	141468	-	80	9.5	5.0	GAGAAtaaa**ATG**		frd.1 [*Enterobacteria* phage ime09] (80/80; 80)	100%	2,00E-50	YP_007004611.1
231	142130	141765	-	121	14.3	5.8	GAAAAcgta**ATG**		frd2 [*Enterobacteria* phage LZ7] (114/121; 121)	94%	2,00E-77	AAA74684.1
232	142423	142175	-	82	9.7	3.8	GGCAAtactgctg**ATG**		e112_255 [*Escherichia* phage e11/2] (76/82; 82)	93%	2,00E-46	YP_009030850.1
233	143456	142548	-	302	33.5	4.8	GAAATaaaa**ATG**	Single-stranded DNA binding, DNA repair, recombination and pre-synthesis	32 [*Enterobacteria* phage ime09] (301/302; 302)	99%	0.0	YP_007004614.1
234	144209	143556	-	217	26.0	9.4	GATTTtctact**ATG**	DNA helicase loader	59 [*Enterobacteria* phage RB14] (217/217; 217)	100%	2,00E-154	YP_002854574.1
235	144544	144206	-	112	12.8	4.5	TTGAActtt**ATG**	Late promoter transcription accessory	33 [*Enterobacteria* phage T4] (112/112; 112)	100%	1,00E-72	NP_049857.1
236	144791	144522	-	89	10.4	5.0	GTGAAta**ATG**	dsDNA binding protein, late transcription	dsbA [*Enterobacteria* phage T4] (89/89; 89)	100%	7,00E-55	NP_049858.1
237	145705	144800	-	306	35.1	8.9	TAGAA**ATG**	Rnase H ribonuclease	rnh [*Enterobacteria* phage T4] (299/301; 305)	99%	0.0	NP_049859.1
238	145822	149694	+	1290	140.4	5.3	GGGAGa**ATG**	Long tail fiber proximal subunit	slur04_00227 [*Escherichia* phage slur04] (1256/1290; 1290)	97%	0.0	CUL02088.1
239	149703	150818	+	371	40.2	5.3	GAGGTatt**ATG**	Hinge connector of long tail fiber	pSs1_00253 [*Shigella* phage pSs-1] (364/371; 371)	98%	0.0	YP_009111061.1
240	150881	151537	+	218	23.3	6.1	GGGCAtaca**ATG**	Small distal long tail fiber subnit	36 [*Enterobacteria* phage RB51] (217/218; 218)	99%	2,00E-154	YP_002854202.1
241	151546	154857	+	1104	118.8	5.8	GAGGActt**ATG**	Large distal long tail fiber subunit	37 [*Enterobacteria* phage AR1] (1050/1103; 1103)	95%	0.0	YP_009168069.1
242	154889	155683	+	264	26.7	7.8	GAGGTttat**ATG**	tail fiber adhesin	ECTP7_01126 [*Escherichia coli* O157 typing phage 7] (252/264; 264)	95%	7,00E-125	AKE45993.1
243	155714	156370	+	218	25.2	7.7	GAGGGtct**ATG**	Holin lysis mediator	t [*Enterobacteria* phage ime09] (218/218; 218)	100%	3,00E-159	YP_007004625.1
244	156643	156371	-	90	10.6	5.4	TATAGtta**ATG**	Anti-sigma 70	asiA [*Enterobacteria* phage T4] (90/90; 90)	100%	4,00E-56	NP_049866.1
245	156808	156656	-	50	5.9	4.6	GATTAtttcga**ATG**		asiA.1 [*Enterobacteria* phage RB51] (50/50; 50)	100%	1,00E-25	YP_002854207.1
246	157083	156805	-	92	10.8	4.5	GGGAAggtct**ATG**	Inhibitor of MrcBC restriction endonuclease	arn [*Enterobacteria* phage RB51] (92/92; 92)	100%	4,00E-57	YP_002854208.1
247	157298	157167	-	43	5.2	8.6	TTGAG**ATG**		arn.1 [*Enterobacteria* phage RB14] (43/43; 43)	100%	1,00E-22	YP_002854588.1
248	157665	157369	-	98	11.4	8.6	GAGGAcctttacta**ATG**		ECML134_254 [*Escherichia* phage ECML-134] (96/98; 98)	98%	2,00E-63	YP_009102729.1
249	158126	157665	-	153	17.9	5.3	GTTTAa**ATG**		arn.3 [*Yersinia* phage PST] (152/153; 153)	99%	1,00E-109	YP_009153859.1
250	158452	158123	-	109	12.7	9.2	GAGGAataaata**ATG**		arn.4 [*Enterobacteria* phage ime09] (109/109; 109)	100%	2,00E-74	YP_007004633.1
251	159098	158463	-	211	23.6	7.8	TAGAAa**ATG**	Activator of middle period transcription	ECML134_257 [*Escherichia* phage ECML-134] (210/211; 211)	99%	3,00E-148	YP_009102732.1
252	159374	159225	-	49	4.9	9.5	AGGAAatcaa**ATG**	Periplasmic protein	MotA.1 [*Enterobacteria* phage T4] (48/49; 49)	99%	3,00E-21	NP_049874.1
253	160699	159371	-	442	50.5	7.7	GTAAAttt**ATG**	DNA topoisomerase subunit	52 [*Enterobacteria* phage RB51] (441/442; 442)	99%	0.0	YP_002854215.1
254	160844	160704	-	46	5.1	8.2	TACTA**ATG**		52.1 [*Enterobacteria* phage RB14] (46/46; 46)	100%	5,00E-21	YP_002854596.1
255	160995	160837	-	52	5.6	4.2	GAAAAT**ATG**	Acridine resistance protein	ac [*Enterobacteria* phage RB14] (52/52; 52)	100%	6,00E-27	YP_002854597.1
256	161589	161083	-	168	18.8	9.6	ATTAAtctatcggccgt**ATG**	Nucleoid disruption protein	e112_280 [*Escherichia* phage e11/2] (166/168; 168)	99%	1,00E-119	YP_009030875.1
257	161817	161602	-	71	8.2	4.2	GTGAAtt**ATG**		slur04_00246 [*Escherichia* phage slur04] (71/71; 71)	100%	2,00E-43	CUL02107.1
258	161933	161826	-	35	4.2	5.3	GTAAAata**ATG**		ndd.2 [*Enterobacteria* phage AR1] (32/35; 36)	91%	2,00E-13	YP_009168086.1
259	162130	161933	-	65	7.5	9.4	GAAAAt**ATG**	Periplasmic protein	ndd.2a [*Enterobacteria* phage ime09] (65/65; 65)	100%	5,00E-38	YP_007004641.1
260	162251	162138	-	37	43	9.5	CACAAa**ATG**	Inner membrane protein	AR1_277 [*Enterobacteria* phage AR1] (36/37; 80)	97%	2,00E-14	YP_009168088.1
261	162857	162495	-	120	14.2	5.4	TACAAcgg**ATG**		Shfl2p275 [*Shigella* phage Shfl2] (117/120; 120)	98%	1,00E-80	YP_004415161.1
262	163396	162839	-	185	21.2	7.7	GAAAGtctaa**ATG**	DNA endonuclease IV	denB [*Enterobacteria* phage RB14] (184/185; 185)	99%	2,00E-135	YP_002854607.1
263	163727	163329	-	132	15.3	4.8	GCGTA**ATG**		RB14ORF272 [*Enterobacteria* phage RB14] (106/109; 109)	97%	1,00E-70	YP_002854608.1
264	163888	163694	-	64	7.5	7.8	GAAAAtt**ATG**		denB.1 [*Enterobacteria* phage RB51] (64/64; 64)	100%	9,00E-36	YP_002854227.1
265	164859	163921	-	312	35.5	5.5	GAAAAtt**ATG**	protector from prophage-induced early lysis	rIIB [*Enterobacteria* phage ime09] (310; 312; 312)	99%	0.0	YP_007004650.1

a Start codon is indicated in boldface; Mach to SD sequence is indicated by underlining; SD position is indicated in uppercase

b The number of identical amino acids/ The total number of amino acids
